# A novel Data and Model Centric artificial intelligence based approach in developing high-performance Named Entity Recognition for Bengali Language

**DOI:** 10.1371/journal.pone.0287818

**Published:** 2023-09-22

**Authors:** Khadija Akter Lima, Khan Md Hasib, Sami Azam, Asif Karim, Sidratul Montaha, Sheak Rashed Haider Noori, Mirjam Jonkman

**Affiliations:** 1 Department of Computer Science and Engineering, Daffodil International University, Dhaka, Bangladesh; 2 Department of Computer Science and Engineering, Bangladesh University of Business and Technology, Dhaka, Bangladesh; 3 Faculty of Science and Technology, Charles Darwin University, Darwin, Northern Territory, Australia; Wright State University, UNITED STATES

## Abstract

Named Entity Recognition (NER) plays a significant role in enhancing the performance of all types of domain specific applications in Natural Language Processing (NLP). According to the type of application, the goal of NER is to identify target entities based on the context of other existing entities in a sentence. Numerous architectures have demonstrated good performance for high-resource languages such as English and Chinese NER. However, currently existing NER models for Bengali could not achieve reliable accuracy due to morphological richness of Bengali and limited availability of resources. This work integrates both Data and Model Centric AI concepts to achieve a state-of-the-art performance. A unique dataset was created for this study demonstrating the impact of a good quality dataset on accuracy. We proposed a method for developing a high quality NER dataset for any language. We have used our dataset to evaluate the performance of various Deep Learning models. A hybrid model performed with the exact match F1 score of 87.50%, partial match F1 score of 92.31%, and micro F1 score of 98.32%. Our proposed model reduces the need for feature engineering and utilizes minimal resources.

## 1. Introduction

Named Entity Recognition (NER) is the task of classifying each token in an input sequence into specific categories based on the context of the sequence. According to its usage, pre-defined categories could be anything, such as name of a person, a place, an organization, a drug [1], a disease [2], a gene [3], time, facility, brand and so on. The availability of NER model is also important for downstream tasks like topic modelling [4], domain specific chatbot building [5], coreference [6] and anaphora resolution [7, 8]. NER is required in the pipeline of any Natural Language Processing (NLP) application that requires automatic text interpretation, such as Information Extraction, Text Summarization [9–11], Question Answering [12, 13] or Machine Translation [14].

Bengali is the sixth most widely spoken language by total number of speakers [15]. Bengali has a large number of vocabulary and research has shown that there are many variations in the formation of Bengali words [16]. Additionally, the diverse use of words complicates the syntactic and semantic structure of Bengali sentences. This kind of diversity makes Bengali NER tasks extremely difficult. Some examples of sequences are included in Table 1 which sheds some light on the Bengali Language and how this makes NER tasks challenging.

**Table 1:** Sample of challenging sequences.

Problem	Sentence
Multiple Meaning	1. মেয়েটির নাম *বকুল* (The girl’s name is *Bakul*) 2. *বকুল* ফুলের সুবাস বেশ মিষ্টি (The fragrance of *Medlar* is so sweet)
	3. *ঢাকা* বাংলাদেশের রাজধানী (*Dhaka* is the capital of Bangladesh) 4. পাত্রটি ঢাকনা দিয়ে *ঢাকা* আছে (The pot is *covered* with a lid)
Idioms	5. আমি *রাবণের চিতায়* জ্বলছি (I am burning in *unquenchable fire*) 6. আমিই সেই *রাবণ* (I am the *Ravana*)
Entity Inflection	7. তার বাড়ি *কুড়িগ্রাম* (His home is in *Kurigram*) 8. লেবুর হালি *কুড়ি টাকা* (Four lemon cost *twenty taka*)
	9. গ্রামটির নাম *আড়াইহাজার* (The name of the village is *Araihazar*) 10. জামাটির মূল্য *আড়াই হাজার* টাকা (The price of the dress is *2500* taka)
	11. সে *এক* এলাহী কান্ড (It was *a* grand arrangement) 12. তার দুই ছেলে, *এক* মেয়ে (He has two sons and *a* daughter)
Multiple Expression	13. কাফি ব্যাংকে *এক লাখ টাকা* জমা রেখেছে (Kafy has deposited *1 lakh taka* in the bank) 14. কাফি ব্যাংকে *১ লক্ষ টাকা* জমা রেখেছে (Kafy has deposited *1 lakh taka* in the bank)
	15. বাংলাদেশে স্বাক্ষরতার হার *৭৪*.*৭০%* (The literacy rate in Bangladesh is *74*.*60%*) 16. বাংলাদেশে স্বাক্ষরতার হার *শতকরা ৭৪*.*৭০ ভাগ* (The literacy rate in Bangladesh is *74*.*60%*)
Expression Similarity	17. সে *১৭৫৭ টাকা* দিয়ে নকশী কাঁথাটি কিনেছে (She has bought the nakshi kantha for *1757 taka*) 18. *১৭৫৭ সালে* পলাশীর যুদ্ধ হয় (The battle of Palashi took place in *1757*)

The capitalization of nouns is one of the most significant advantages of English NER. Unlike English, the Bengali language does not have the concept of capitalization. In addition, the same word may have multiple meanings, based on the context of the word sequence in Bengali. The first two pairs of sentences in Table 1 represent the problem of *multiple meanings*. In sentence 1, the token বকুল refers to a person’s name, whereas in sentence 2 the same token is used to refer to the name of a flower. In sentence 3, the token ঢাকা is used as the name of a city while in sentence 4 the same token indicates that something is covered. The use of idiom makes Bengali text incomprehensible for machines. In sentence 5, the phrase রাবণের চিতা means unquenchable fire. However, in general usage, রাবণ and চিতা have different meanings. For example, in sentence 6, the token রাবণ is used to refer to a person. Bengali also has many inflected words. In sentence 7, কুড়ি added with the affix গ্রাম forms a new token কুড়িগ্রাম indicating a place name. In sentence 8 the token কুড়ি combined with another token টাকা refers to a monetary expression. The root word কুড়ি is inflected. In sentence 9, the token আড়াইহাজার is a place name but when there is a space between আড়াই and হাজার, they form a monetary expression as in sentence 10. In this case, both root words আড়াই and হাজার are inflected. In sentence 12, the token এক indicates a quantitative expression whereas in sentence 11 the same token does not. Another challenge for Bengali NER is the issue of multiple expressions. In Bengali, it is possible to convey a word or a phrase in a variety of ways. In sentence 13 and 14, the phrase 1 lakh taka is expressed as এক লাখ টাকা and ১ লক্ষ টাকা respectively. They both have the same meaning and indicate a monetary expression. In sentence 15 and 16, the token 74.70% is expressed as ৭৪.৭০% and শতকরা ৭৪.৭০ ভাগ respectively. Again, the meaning is the same. There is a similarity between expressions for percentages, quantities, and monetary entities. Within the context of sentence 17, the token ১৭৫৭ belongs to a monetary expression. In sentence 18, however, the same token refers to a time expression.

The early stage research for Bengali NER mainly focused on statistical and machine learning approaches, such as Hidden Markov Model [17, 18], Conditional Random Fields [19, 20], Support Vector Machines [21], Maximum Entropy [22], Multi Engine method [23–25], and Margin Infused Relaxed algorithm [26]. However, these studies required hand-crafted features like POS tagger, Lexicons, etc. to boost model performance. With the advancement of Deep Learning (DL) technology, it is now possible to produce human-level accuracy in NER tasks while avoiding dependence on hand-crafted features. To address the challenges described above, we have developed Deep Learning models which utilize minimal resources to perform NER task for a low-resource language like Bengali. This research emphasizes both Model and Data-Centric AI concepts in order to obtain higher accuracy. Model-Centric AI is concerned with selecting the right model architecture, while Data-Centric AI is concerned with improving the quality of the dataset. In recent years, some DL based NER systems [27–30] have been developed for Bengali NER. Studies prioritizing only Model-Centric AI were not able to achieve competitive accuracies for Bengali to NER systems for other languages. Karim *et al*. [29] and Ashrafi *et al*. [30]. trained NER models on same dataset developed by Karim *et al*. [29]. However, the systems could not achieve high accuracy in Bengali NER compared to English and Chinese NER due to a lack of quality of the data. Data imbalance is a common issue for NER. With some exceptions [30], previous studies have ignored data imbalance issues for Bengali NER. It is important to handle imbalance in order to control training bias. We have therefore applied two common data imbalance handling techniques in this research. Key contributions of this research are as follows:

- For the first time, Data and Model-Centric AI concepts are combined for Bengali NER, resulting in competitive accuracies to English and Chinese NER.
- We proposed a method for developing the NER dataset and developed a unique dataset. Our NER dataset is the biggest Bengali NER dataset to date, containing 77,277 sentences and 10,05,791 tokens.
- We performed five major model-centric experiments to develop a robust NER system for morphologically complex language like Bengali.
- We developed a robust model for a low-resource language like Bengali utilizing our NER dataset and pretrained word embeddings only.

## 2. Literature review

Several methods have been proposed by researchers for Named Entity Recognition. In this section, we discuss some previous works on NER for Bengali and other languages. Ekbal *et al*. [17] introduced a Hidden Markov Model (HMM) based NER system for Bengali. The authors worked with an annotated Bengali news corpus dataset which was developed from the archive of a widely read Bengali newspaper. The system was trained with 150 thousand word forms. After a 10-fold cross validation, the average values of Precision, Recall, and F1-Score were 79.48%, 90.2%, and 84.5% respectively. Afterwards, this HMM based system was also trained and validated on 27,151 Hindi word forms. The system recorded the average Precision, Recall, and F1 scores for Hindi of 74.6%, 82.5%, and 78.35% respectively.

Other researchers [21] employed Support Vector Machine (SVM) for training their system with the same dataset. Their model achieved the Precision, Recall and F1 score of 89.4%, 94.3%, and 91.8% respectively. The same dataset was also used for training a CRF based Bengali NER system [19], where experimental results of a 10-fold cross validation yielded Recall, Precision and F1 scores of 93.8%, 87.8%, and 90.7% respectively.

To solve the challenge of automatic detection of the Named Entities (NE) from Bengali text, Chaudhuri *et al*. [25] proposed a three stage approach combining NE dictionary, rules, and co-occurrence statistics. The system was trained with 70 thousand tokens and tested with 20 thousand tokens, achieving an average Precision, Recall, and F-score of 85.50%, 94.24%, and 89.51% respectively.

A Maximum Entropy (ME) based NER system for Bengali was developed and trained with 1,22,467 tokens [22]. The system obtained average Precision, Recall, and F1 scores of 82.63%, 88.01%, and 85.22% respectively.

A new Bengali NER system was proposed by [24] combining the outputs produced from three classifiers (Maximum Entropy, Conditional Random Field, and Support Vector Machine). Employing a training set of 150 thousand word forms, the model obtained overall Precision, Recall, and F1 scores of 83.61%, 87.11%, and 85.32% respectively.

A multi-engine approach using weighted voting technique was proposed by Ekbal *et al*. [23]. Training with 150 thousand word forms, the system demonstrated Precision, Recall, and F1 scores of 90.63%, 93.98%, and 92.28% respectively.

Independent and dependent features for the Bengali NER tasks were recognized in order to develop a system based on the Margin Infused Relaxed Algorithm [26]. The system yielded a F1 score of 89.13%.

Parvez *et al*. [18] developed a HMM based NER model using a POS tagger containing 56,196 Bengali words. Their model was trained with only one sentence of 21 tagged words and tested with two sentences, achieving Precision, Recall and F1 scores of 85.7%, 94.7%, and 90% respectively.

Banik *et al*. [27] applied a Gated Recurrent Unit (GRU) based Deep learning model to develop a Bengali NER system on a manually annotated dataset. The system had testing accuracy, and F1 scores of 93.31% and 69.42% respectively.

A partial string matching approach, based on Breadth First Search (BFS), was proposed by Ibtehaz *et al*. [31] to identify NE’s from an unstructured Bengali text corpus.

Chowdhury *et al*. [20] used a combination of various features with a CRF based model for Bengali NER and applied it to their dataset. The model was trained with 1,510 sentences and tested with 427 sentences. The model obtained Precision, Recall, and F1 scores of 65%, 53%, and 58% respectively for exact matches.

An overview of previously introduced methods for Bengali NER was provided by [28] and some Deep Learning models were further explored. Their data source was a renowned newspaper with a total number of 96,697 tokens in the dataset, which was divided in 67,554 for training and 29,143 for testing. The Bidirectional Gated Recurrent Unit (BGRU) based model gained the F1 score of 72.66%.

Karim *et al*. [29] proposed a Bengali NER system which utilized Densely Connected Network (DCN) in collaboration with Bidirectional LSTM (BiLSTM) and word embedding. They also developed a dataset containing 9,83,663 tokens. The experiments were conducted by comparing two word embedding models (Word2Vec and Glove) and two character level feature extraction models (CNN and DCN). The model achieved Precision, Recall, and F1 scores of 68.95%, 58.62%, and 63.37% respectively.

In a recent study, Ashrafi *et al*. [30] performed the Bengali NER task using Word2Vec and Bidirectional Encoder Representations (BERT) models. They explored various deep learning models and proposed a cost sensitive learning method to address the class imbalance in the data. Their best performing model BERT+BiLSTM+CRF+CW obtained a macro F1 score of 65.96%, a micro F1 score of 90.64% and a Message Understanding Coreference (MUC) F1 score of 72.04%.

Other researchers have looked at NER systems suitable for different languages. Santos *et al*. [32] proposed a language-independent system that utilized both word and character level features to perform sequence labeling tasks. They evaluated results for Spanish and Portuguese NER. Their model performed best in the Spanish NER task by recording Precision, Recall, and F1 scores of 82.21%, 82.21%, and 82.21% respectively. For Portuguese NER, the Precision, Recall, and F1 scores were 73.98%, 68.68%, and 71.23% respectively.

Chiu *et al*. [33] proposed a novel neural network based hybrid architecture consisting of BiLSTM, CNN, word embedding, capitalization feature, and lexicons for capturing word and character-level features. The proposed model achieved an F1 score of 91.62% on the CONLL-2003 dataset and 86.28% on the OntoNotes 5.0 dataset.

Ma *et al*. [34] combined BiLSTM, CNN, and CRF to capture word and character-level representations. Evaluating the model for part-of-speech (POS) tagging and Named Entity Recognition (NER) in two datasets, the model achieved F1 scores of 97.55% for POS tagging and 91.21% for NER.

A neural reranking NER system based on Long Short Term Memory (LSTM) and CNN structures was proposed by Yang et *al*. [35]. Their system outperformed all the previously existing models with an F1 score of 91.62%.

Lexical features are useful for neural network based NER systems. Ghaddar *et al*. [36] utilized a single Bi-LSTM layer at word level. In order to combine words and entity types into a one-dimensional vector space, annotated data was produced to train and develop the model. They achieved F1 scores of 91.73%, and 87.95% for the CONLL-2003, and OntoNotes 5.0 dataset respectively.

## 3. Dataset preperation

The history of Bangla Natural Language Processing (BNLP) research spans over a decade. Initially, the research progressed slowly due to a lack of resources. There has been a big change in this regard in the few years though. Many attempt to make benchmark datasets for BNLP are currently ongoing. As a consequence, there are now datasets for Bangla Abstractive Text Summarization [37], Question Answering [38], Authorship Classification [39], and Machine Translation [40]. These studies have shown that deep learning models trained on a large amount of data achieve a high level of accuracy. The amount of data required is determined by the complexity of the task. NER is a complex sequence labeling task and datasets tend to be skewed towards the non-named entity class. Since only proper training of the model can ensure reliable accuracy, we need a large amount of quality data for training. Although there are high quality NER datasets for English, Chinese, German, and other languages there is still a lack of high quality datasets for Bengali. Our work therefore begins with the development of a benchmark dataset for Bengali Named Entity Recognition.

**Fig 1:**
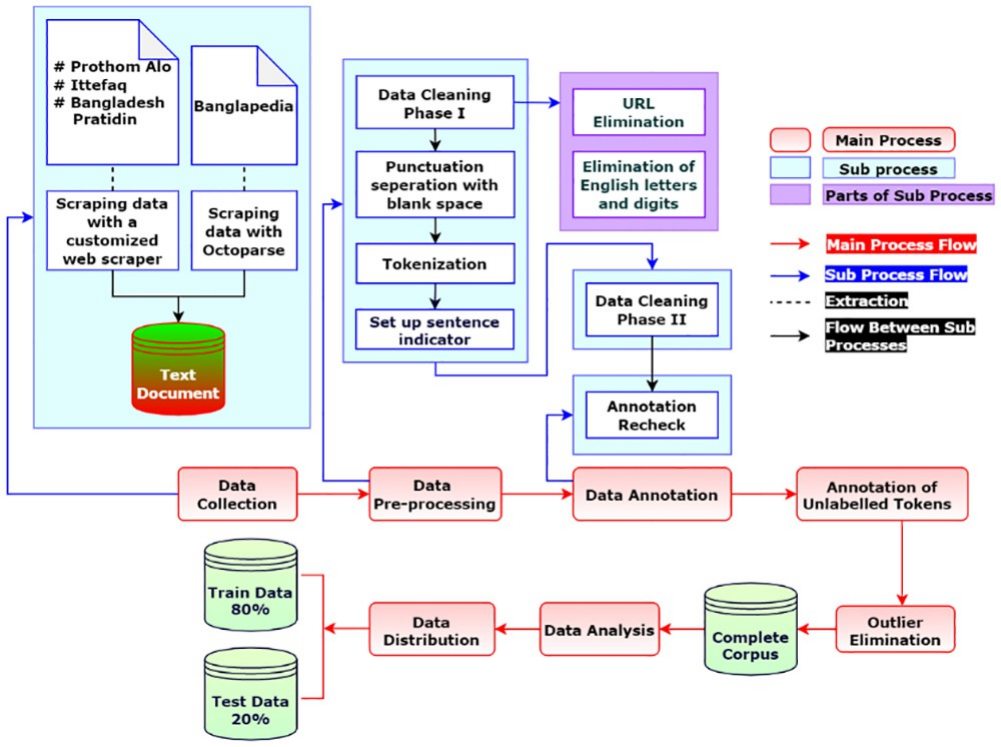
Complete process of dataset preparation.

Fig 1 shows the development lifecycle of our dataset. The diagram illustrates seven main processes (red boxes) along with their sub-processes (white boxes). In the following subsections, all of these major processes are described in depth.

### 3.1 Data collection

Dataset preparation starts with the data collection. At present, Bangladesh is rapidly progressing towards digitalized blogs, articles and news with a variety of words and sentences. In this regard, online platforms can be considered as a good source of the Bangla text corpus. We chose Banglapedia along with other popular Bangla news portals such as Prothom Alo, Bangladesh Pratidin and Ittefaq to collect the data.

Scraping is the most efficient way to collect a large number of news articles and other items in a short time. Considering the architecture of the websites mentioned above, we used two different scrapers which requires limited resources and memory space. We constructed a scraper with the widely used *Python* web scraping libraries *BeautifulSoup* and *Requests*. On the other hand, Octoparse, a versatile web crawling tool, was used to extract Bangla text from Banglapedia web pages. Table 2 shows the total number of articles collected from each website. After successful scraping, we collected 1005 articles in a number of different categories: National, International, Science, Health, Sports, Editorial, Entertainment, Economy, Education, and Politics, in order to avoid bias for in any particular area.

**Table 2:** Article statistics.

Website	Article Count
Prothom Alo	300
Bangladesh Pratidin	155
Ittefaq	300
Banglapedia	250
**Total**	**1005**

### 3.2 Data pre-processing

The data that were collected from several web sources contained garbage information. For instance, some texts had hyperlinks to other websites. Moreover, as English is the second language in Bangladesh, English words and numbers can also be found in the texts. The raw data has to go through two Data Cleaning or garbage elimination phases. We filtered out anomalies like URLs, English letters and digits by utilizing the popular Python module *Regular Expression* in the initial phase of data cleaning.

It was also noticed that punctuation marks were attached to words in Bangla text. It was therefore necessary to separate each punctuation mark from word prior to tokenization. All punctuation marks were separated from words by a space on *Notepad++*. Table 3 presents an example of a sentence with four commas and one full stop in its real structure. We added an extra space before all four commas and the full stop to separate the punctuation marks from the words.

**Table 3:** Sentence structure conversion by separating punctuation.

**Before Conversion**	অন্ন, বস্ত্র, বাসস্থান, শিক্ষা, চিকিৎসা হলো জীবনধারণের মৌলিক উপকরণ। (Food, clothing, shelter, education, medicine are the basic necessities of life.)
**After Conversion**	অন্ন, বস্ত্র, বাসস্থান, শিক্ষা, চিকিৎসা হলো জীবনধারণের মৌলিক উপকরণ । (Food, clothing, shelter, education, medicine are the basic necessities of life.)

With the help of the Python string manipulation function *Split*, we subsequently split the strings at the blank spaces and placed each word on a new line. In Bengali, a full stop (।) or question mark (?) or exclamation mark (!) is usually used at the end of a sentence. We used this to distinguish individual sentences, and added a new blank line at the end of each sentence. Table 4 shows the following sentence after tokenization and set up sentence indicator: কুটিরশিল্প বাংলাদেশের ঐতিহ্যবাহী একটি শিল্প । (Home-craft is a traditional artistry of Bangladesh.)

**Table 4:** Sentence after tokenization and set up sentence indicator.

কুটিরশিল্প
বাংলাদেশের
ঐতিহ্যবাহী
একটি
শিল্প
।

### 3.3 Data annotation

We followed the *IOB2* format to annotate all sentences. The *IOB2* format is used to tag the tokens in a chunking task, with the *I-tag* indicating a token within a chunk, the *O-tag* indicating the tokens outside of the chunk, and the *B-tag* indicating a token at the start of a chunk. Table 5 includes a detailed description of this tagging style. The annotation of a person’s name by the tag *PER* is misleading, as a person’s name can be formed of multiple parts. To avoid confusion, the *IOB2* tagging scheme implies that a person’s name begins with *B-PER*, and *I-PER* tags the inside the same chunk. A similar technique was applied to label Location, Organization, Quantity, Percentage, and Currency entities. Tokens which do not belong to a chunk were annotated with *O-tag*. During annotation, we considered only the name of an individual as a *Person* entity. The name of a country or the name of a place within a country was labelled as a *Location* entity. *Organization* entities include all types of charities, educational institutions, business organizations, government, and non-government organizations. Monetary and percentage expressions were labelled as *Currency* and *Percentage* entities, respectively. We considered mass nouns, unit nouns, ordinal numbers, definite and indefinite numbers as *Quantity* entities. It was observed during annotation that some sentences contained offensive or personal information. Such sentences were removed in the second phase of data cleaning. Initially, the annotation process involved three human annotators. It was noticed that this caused an inconsistency in tagging due to the fact that different people have different viewpoints. Moreover, Bengali is rich in words and the meaning of a word may vary depending on the context. A Bangla language expert therefore re-checked and finalized all the tags, making corrections where necessary to minimize inconsistency and prioritizing the majority voting techniques. The **Acknowledgments** section contains information on the qualifications and experience of the individuals who contributed to the annotations. The finalization of the tags from the first three phases is demonstrated through a sample file of annotation phases (https://github.com/raktim52/Raktim_52/blob/main/data_annotation_phases.xlsx). The final version of the dataset can be accessed from the publicly available GitHub repository *Raktim_52* for further research (https://github.com/raktim52/Raktim_52). Table 6 represents the following sentence from our dataset: লক্ষ্মণ সেনের পিতার নাম বল্লাল সেন । (Lakshman Sen’s father’s name is Ballal Sen.). In this sentence, *Lakhsman Sen* and *Ballal Sen* are the chunks of *Person* entity, whereas the rest of the tokens are not part of any chunk.

**Table 5:** Tagging scheme.

Tag	Example	Explanation
*B-PER*	*ব্যোমকেশ* বক্সী Byomkesh Bakshi	Tags the starting of a person name
*I-PER*	ব্যোমকেশ *বক্সী* Byomkesh Bakshi	Tags the inside of a multi-word person name
*B-LOC*	*উত্তর* সেহাচর North Sehachar	Tags the starting of a location name
*I-LOC*	উত্তর *সেহাচর* North Shehachar	Tags the inside of a multi-word location name
*B-ORG*	*ঢাকা* কলেজ Dhaka College	Tags the starting of an organization name
*I-ORG*	ঢাকা *কলেজ* Dhaka College	Tags the inside of a multi-word organization name
*B-QTY*	*১০০* টন 100 tons	Tags the starting of a quantity indicating phrase
*I-QTY*	১০০ *টন* 100 tons	Tags the inside of a quantity indicating phrase
*B-CUR*	*১০০* টাকা 100 taka	Tags the starting of a currency indicating phrase
*I-CUR*	১০০ *টাকা* 100 taka	Tags the inside of a currency indicating phrase
*B-PCT*	*শতকরা* ৫০ ভাগ 50%	Tags the starting of a percentage indicating phrase
*I-PCT*	শতকরা *৫০ ভাগ* 50%	Tags the inside of a percentage indicating phrase
O	সম্পূর্ণ Complete	Tags punctuations and anything except the above mentioned categories

**Table 6:** Sample of NER dataset.

লক্ষ্মণ	B-PER
সেনের	I-PER
পিতার	O
নাম	O
বল্লাল	B-PER
সেন	I-PER
।	O

It was observed during annotation that some sentences contained offensive or personal information. Such sentences were removed in the second phase of data cleaning. Initially, the annotation process involved more than one human annotator. It was noticed however that this caused an inconsistency in tagging due to the fact that different people have different viewpoints. Moreover, Bengali is rich in words and the meaning of a word may vary depending on the context. A Bangla language expert therefore re-checked and finalized all the tags, making corrections where necessary to minimize inconsistency. Table 6 represents the following sentence from our dataset: লক্ষ্মণ সেনের পিতার নাম বল্লাল সেন । (Lakshman Sen’s father’s name is Ballal Sen.). In this sentence, *Lakhsman Sen* and *Ballal Sen* are the chunks of *Person* entity, whereas the rest of the tokens are not part of any chunk.

### 3.4 Annotation of unlabeled tokens

Occasionally tokens were left unlabelled. It is important to detect any tokens that were not labelled. Algorithm 1 describes the process of finding these unlabelled tokens. We need the line numbers of the unlabelled tokens. First, the data file is read by encoding it in ‘utf-8’. The variable *lineNo* is initialized to 0. This keeps track of the line number. For each line, the *words* variable keeps track of the strings of that line and the *wordList* variable contains a list of strings created by splitting the line into blank spaces. Each line can have a maximum of two strings: Token and Label. As our data file uses a blank line at the end of each sentence, the *wordList* variable can be an empty list. It increases the *lineNo* by 1 for both empty and non-empty lists. When the list is non-empty, it also checks whether the length of the *wordList* is equal to 1 or not. If the length of the *wordList* is equal to 1, it prints the *lineNo* so that we can find the unlabeled token’s line number and label it. While searching for the *wordList* of length one, it was discovered that some sentence indicator lines had been tagged by mistake. We removed the tags from those lines.

**Algorithm 1** Finding Unlabelled Tokens

**BEGIN**

1.  **Input:** read the data file

2.  lineNo = 0

3.  **FOR** line in input **DO**

4.   words **=** line from the input

5.   wordList = make a list of words

6.   **IF** wordList is not empty **THEN**

7.    lineNo + = 1

8.    **IF** size length of list is equal to **THEN**

9.     **PRINT →** lineNo

10.    **ENDIF**

11.   **ENDIF**

12.   **ELSE**

13.     lineNo + = 1

14.   **ENDELSE**

15.  **ENDFOR**

**END**

### 3.5 Outlier elimination

As all the tokens were manually annotated, there may be some human errors. All the tags in our dataset were written in capital letters. However, we may sometimes mistakenly use lower case letters instead. For example, we needed to tag a token ’O’ instead of ’o’. To correct this type of error, it is important to know all the unique tags in the dataset. Algorithm 2 provides the procedure for extracting unique tags from the dataset. Algorithm 2 follows the same procedure as algorithm 1 to read the data and convert each line into a list. An empty list ‘*tags’* is declared for subsequent use. When the list is non-empty, it adds the tag to the list *tags*, thereby storing the tags of all the tokens in a list. Another empty list ‘*uniqueTags’* is declared to find all the unique tags in the *tags* list. For each tag, it checks whether the tag is already present in *uniqueTags* or not. If the tag is not in *uniqueTags*, it adds the tag to the list *uniqueTags*. By printing all the unique tags, it can be seen if any outliers exist or not. The next step is finding the line numbers of these tags. Algorithm 3 follows the same procedure as algorithm 1 to read the data, keep track of the line numbers and convert each line into a list. When the list is non-empty, it checks whether the index 1 of *wordList* is matched with an outlier or not. If it matches, it prints the *lineNo* so that we can find the line number and correct the tag.

**Algorithm 2** Finding Unique Tokens

**BEGIN**

1.  **Input:** read the data file

2.  tags = []

3.  **FOR** line in input **DO**

4.   words **=** line from the input

5.   wordList = make a list of words

6.   **IF** wordList is not empty **THEN**

7.    tags = tags + [wordList[1]]

8.   **ENDIF**

9.   **ENDFOR**

10.   uniqueTags = []

11.   **FOR** tag in tags **DO**

12.    **IF** tag **NOT IN** uniqueTags **THEN**

13.     uniqueTags = uniqueTags + [tag]

14.    **ENDIF**

15.  **ENDFOR**

16.  **PRINT →** uniqueTags

**END**

**Algorithm 3** Finding Outliers

**BEGIN**

1.  **Input:** read the data file

2.  lineNo = 0

3.  **FOR** line in input **DO**

4.   words **=** line from the input

5.   wordList = make a list of words

6.   **IF** wordList is not empty **THEN**

7.    lineNo + = 1

8.    **IF** wordList[1] = = ‘Outlier’ **THEN**

9.     **PRINT →** lineNo

10.    **ENDIF**

11.   **ENDIF**

12.   **ELSE**

13.     lineNo + = 1

14.   **ENDELSE**

15.  **ENDFOR**

**END**

### 3.6 Data analysis

**Fig 2:**
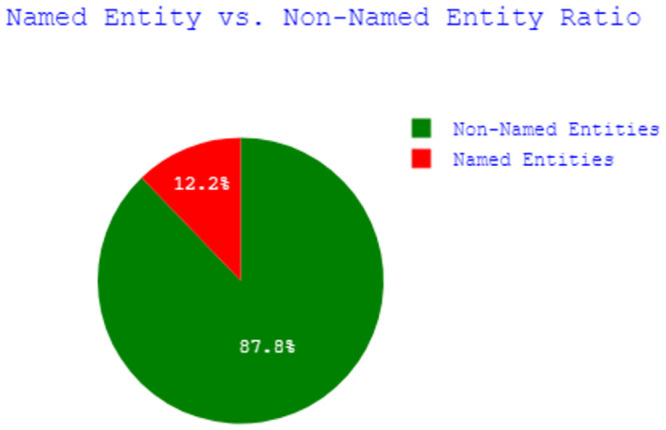
Named entity vs. non-named entity ratio.

**Fig 3:**
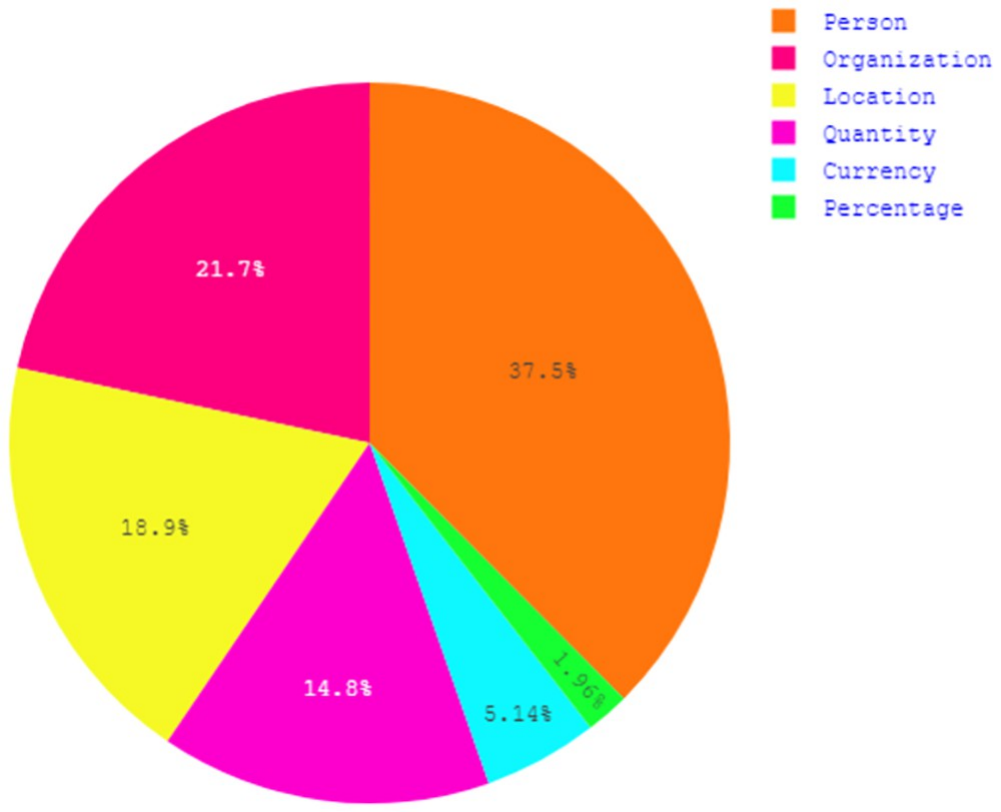
Ratio of named entities.

**Fig 4:**
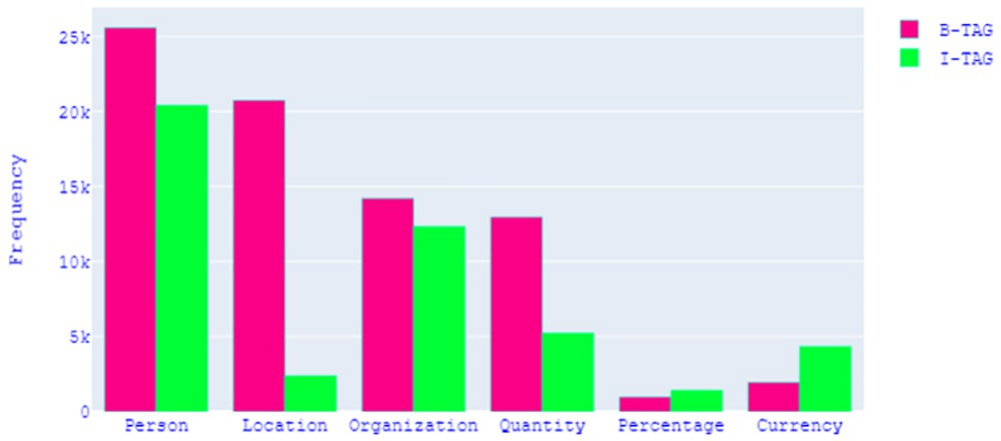
Frequency of named entities.

After completing the dataset, it is important to know about the pros and cons of the dataset. Data analysis was done to uncover the underlying structure of the dataset, revealing important patterns and relationships that were not immediately apparent. Table 7 gives an overview of the content of our dataset. After the two-phase garbage elimination, 77,277 sentences remained for our research, where the length of the sentences varied from 3 to 145. Our dataset contains more than *1 million* tokens, with approximately *65 thousand* of them being unique. The pie chart shown in Fig 2 depicts the ratio of named entities to non-named entities for our dataset. It demonstrates that the dataset is highly biased towards Non-Named entities. As shown in Fig 3, the proportions of Person, Organization, Location, Quantity, Currency, and Percentage entities are 37.5%, 21.7%, 18.9%, 14.8%, 5.14%, and 1.96% respectively. The grouped bar chart in Fig 4 illustrates the frequency of each Named Entity, with pink-coloured bars representing *B-tag* and green-coloured bars representing *I-tag*. Analysis of the Named Entity distribution for the entire dataset shows that there is a higher frequency of the beginning parts of the Person, Location, Organization, and Quantity entities than of the inside part. For the Percentage and Currency entities this is the other way around.

**Table 7:** Dataset statistics.

#	Frequency
Total number of sentences	77,277
Total number of tokens	10,05,791
Total number of unique tokens	64,958
Sentence length	3–145

### 3.7 Data distribution

We split our dataset into a training set and a testing set. The training set contains 80% of the tokens, and the testing set contains the remaining 20%. There are 61,641 sentences in the training set and 15,636 in the testing set. Table 8 includes the tag distribution of each entity for both the training and testing set. It is obvious that there are a large number of samples with the tag ‘O’ in the dataset. Some tags, such as I-LOC, B-PCT, I-PCT, B-CUR have very few samples, which may complicate the learning process of the models.

**Table 8:** Train and test data distribution.

Entity	Tag	Training Data	Testing Data
Person	B-PER	21,220	4,356
	I-PER	17,074	3,344
Location	B-LOC	16,016	4,723
	I-LOC	1,990	406
Organization	B-ORG	11,870	2,328
	I-ORG	10,121	2,212
Quantity	B-QTY	10,540	2,416
	I-QTY	4,130	1,096
Percentage	B-PCT	7,28	253
	I-PCT	1,075	348
Currency	B-CUR	1,605	342
	I-CUR	3,657	692
Others	O	7,04,611	1,78,638
		**Total**	**Total**
		8,04,637	2,01,154

## 4. Problem definition

Assuming that a sentence consists of *n* number of words. If the sentence is represented as a set *s*, then *s = {w*_*1*_, *w*_*2*_, *w*_*3*_,*…*., *w*_*n*_*}* where each word is considered as a token, the required output is such that *t = {t*_*1*_, *t*_*2*_, *t*_*3*_,*…*., *t*_*n*_*}*. Here, *t*_*i*_ *∈ {B-PER*, *I-PER*, *B-LOC*, *I-LOC*, *B-ORG*, *I-ORG*, *B-QTY*, *I-QTY*, *B-PCT*, *I-PCT*, *B-CUR*, *I-CUR*, *O}*. The context of the sentence is required to be considered when tagging each token present in that sentence.

## 5. Models

**Fig 5:**
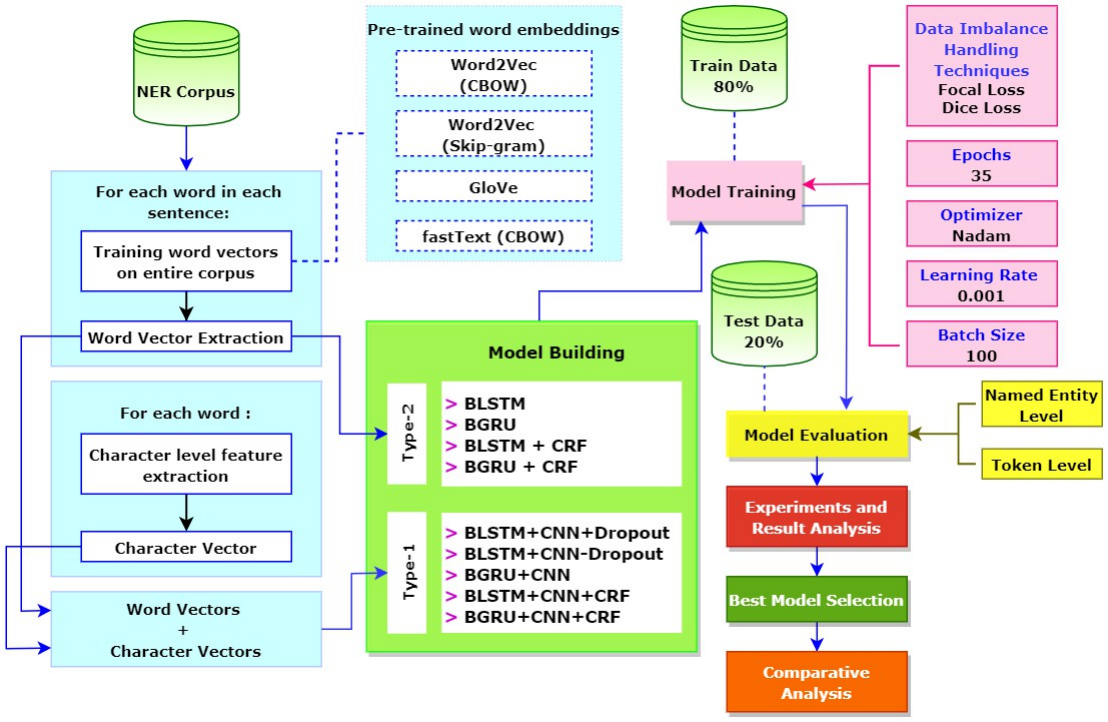
Workflow diagram.

This research involves the following steps: Dataset preparation, Model Building, Model Training, Model Evaluation, Experiments and Result Analysis, Best Model Selection, and Comparative Analysis. Fig 5 depicts a conceptual diagram showing this.

After developing the NER dataset, the aim of this research is to explore various neural networks based models. These can be categorized into two types. Type-1 models utilize both word and character level features. In contrast, Type-2 models utilize only word-level features. The performance of each model is examined with four different word embeddings. Analyzing the experimental results, we proposed a model. The following subsections describe the details of both types of models as well as the components required to build them.

### 5.1 Model overview

**Fig 6:**
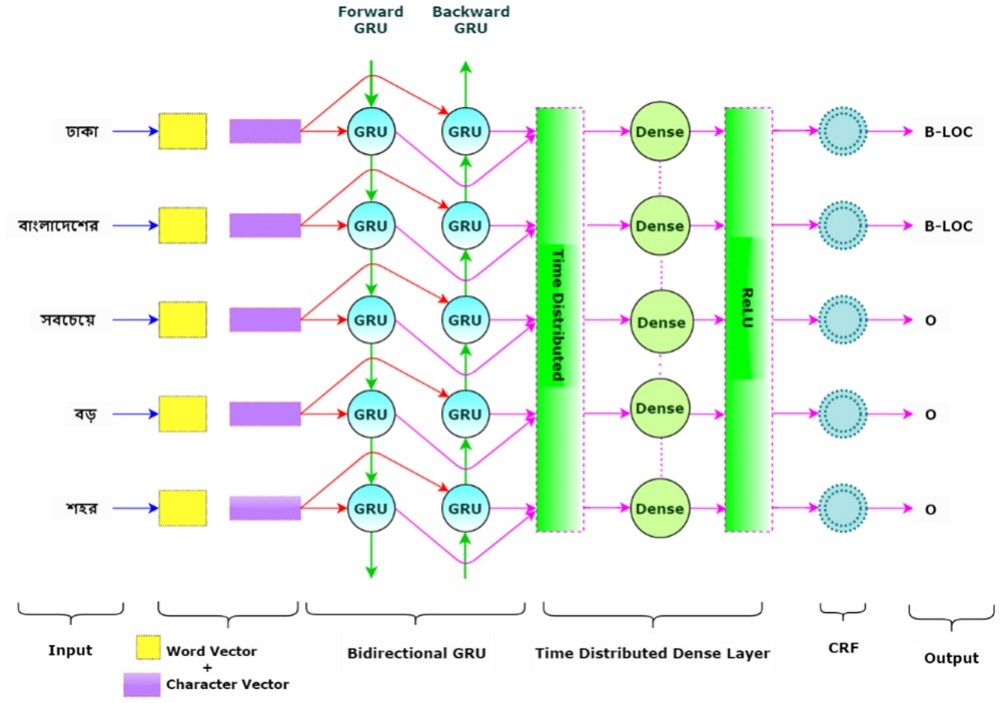
Type-1 model architecture.

The model takes sentences from the dataset as input. For each sentence, word-level feature representations are extracted from the pre-trained non-contextualized word embeddings (Word2Vec, GloVe, and fastText). Character level features are extracted using a Convolutional Neural Network. The concatenation of word and character representations is sent to a Bidirectional Gated Recurrent Unit (BGRU) neural network. Bidirectional Long Short-Term Memory (BLSTM) is sometimes employed instead of BGRU. Only one model (BLSTM + CNN + Dropout) recurrent dropout in this layer. Dropout is avoided for the other four models due to the potential reduction in performance. The hidden states generated with the BGRU or BLSTM layer are fed into a time-distributed dense layer of 13 units, using the activation function softmax. For experiments with CRF, the outputs produced from the BGRU or BLSTM layer are sent to a time-distributed dense layer of 50 units using the activation function Rectified Linear Unit (ReLU), which is followed by a CRF layer. The CRF layer provides the output for each token. Fig 6 depicts an overview of type-1 models. To illustrate the concept in a simple way, only one hidden state is displayed in the BGRU and Dense layers.

**Fig 7:**
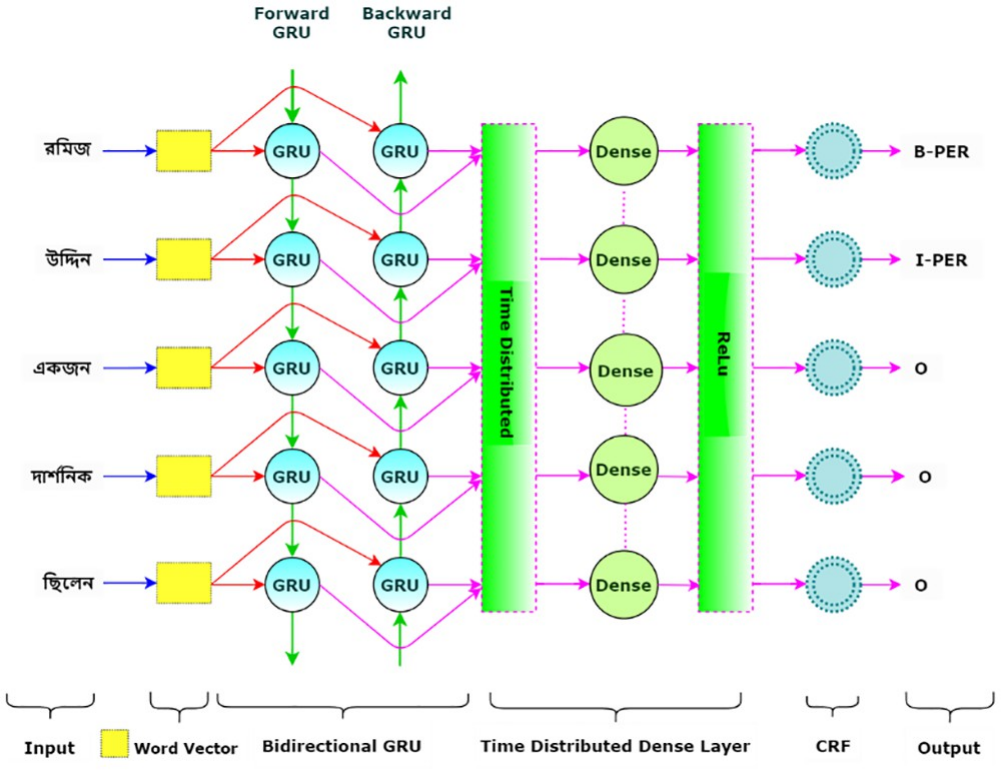
Type-2 model architecture.

Fig 7 gives an overview of type-2 models. The architecture of type-2 models is similar to that of type-1 models, with one significant difference. Type-2 models only utilize word representations and completely exclude character-level features. Type-2 models send only word representations to the BGRU or BLSTM layer instead of the concatenation of word and character representations. The size of the hidden states and other parameters for the BGRU or BLSTM, Dense and CRF layers are the same as in type-1 models. Type-2 models completely avoid dropout. Both types of models are inspired by the architecture of Chiu *et al*. [33], Ma *et al*. [34].

### 5.2 Pretrained word embeddings

In natural language processing, word embedding is an extensively used technique. Word embedding is the process of converting a word or phrase into a numeric vector, capturing the syntactic and semantic meaning of the word, as well as its relationship with other words in a document. As a result, words with similar meanings have a close encoding to each other. This method is adopted to improve the efficiency of neural networks in understanding human languages. The use of one-hot encoding representation for 64,958 unique words in our dataset would be inefficient, increasing the dimensionality. It is also quite impossible to include all potential examples of each entity type in the training data. Word embedding provides an effective solution to these problems. Let, our training data contain the following sequence: তিনি বর্তমানে গুগলে কর্মরত আছেন। (Currently, she is working at Google.). Here, *Google* is annotated as *B-ORG*. This is needed to make a prediction for the following sequence: তিনি বর্তমানে আমাজনে কর্মরত আছেন। (Currently, she is working at Amazon.). Even though our train data do not contain *Amazon*, our model can tag this token as *B-ORG* utilizing word embedding as the values for *Amazon* and *Google* in vector space are closer to each other. As the findings of Chiu *et al*. [33] and Karim *et al*. [29] show, the performance of NER tasks varies depending on the word embeddings used. We have trained our models using four different publicly accessible pre-trained word embeddings. The following subsections include the details of each word embedding.

#### 5.2.1 Word2Vec

Mikolov *et al*. [41] first introduced two variants of Word2Vec: CBOW and Skip-gram. The CBOW model considers the context of adjacent words when predicting the current word. The Skip-gram model predicts adjacent words based on the current word. Assume that a sentence consists of five words. If the sentence is presented as a set, then *s = {w*_*1*_, *w*_*2*_, *w*_*3*_, *w*_*4*_, *w*_*5*_*}* where w_3_ is the current word. To predict *w*_*3*_, the CBOW model takes the context of preceding words (*w*_*1*_, *w*_*2*_) and the future words (*w*_*4*_, *w*_*5*_) into account. The Skip-gram model predicts the *w*_*1*_, *w*_*2*_, *w*_*4*_, *w*_*5*_ using *w*_*3*_. Both models are dependent on the local context information of the words.

In this study, we have used the 300-dimensional Word2Vec (CBOW) which was created by Alam *et al*. [42] for another sequence labeling task called *POS tagging*. The vocab size of the model is 436,126. We have also experimented with the 300-dimensional Word2Vec (Skip-gram) released by Sarker *et al*. [43] which was trained on the Bengali Wikipedia Dump Dataset. *20 million* tokens were used to train the model and it has a vocabulary of 11,71,011.

#### 5.2.2 Global Vectors (GloVe)

Pennington *et al*. [44] pioneered a word embedding approach that captures global information in the form of a word co-occurrence matrix and integrates local information to generate word vectors. This approach resulted in an impressive performance in a variety of sequence labeling tasks. We have utilized the 300-dimensional GloVe which was developed by Sarker *et al*. [43]. The GloVe model was trained on Wikipedia and news article corpus. *39 million* tokens were used to train the model. The model has a vocabulary of 1,78,152.

#### 5.2.3 FastText (CBOW)

Bojanowski *et al*. [45] proposed a word embedding technique that learns morphology by integrating sub-word components. In this method, each word is represented as a collection of character n-grams. Each character n-gram has a corresponding vector representation. The sum of all of these vector representations is used to represent a word. This model is an extension of the Skip-gram model proposed by Mikolov *et al*. [41]. Grave *et al*. [46] proposed a variant of fastText (Skip-gram) which is capable of capturing positional information. Let, *s* be *{w*_*-n*_,*…*., *w*_*-1*_,*…*., *w*_*1*_,*…*., *w*_*n*_*}*. Here, *s* represents a sequence of *n* words. To predict a target word *w*_*0*_, the model considers the positional information, character n-grams, and context of surrounding words. The following equation gives a vector representation of this context:

V=∑i=-ni≠0nci⊙vwi (1)

Here, *V* is the average of the vectors relating to the words in this context, *c*_*i*_ are the vectors corresponding to each position in the window, and ⊙ is element-wise multiplication [46]. $v_{w_{i}}$ are the word vectors where each *w*_*i*_ is obtained by adding the vector representation of all the character n-grams that appear in it.

As Bengali is a morphologically enriched language, we have used the 300-dimensional fastText (CBOW) model released by Grave *et al*. [46]. The model was trained on Common Crawl and Wikipedia. The length of character n-grams is five.

As mentioned earlier, there are 64,958 unique tokens in our dataset. For the unique tokens, the number of tokens found with each word embedding is shown in Table 9. As it can be observed, the percentage of matches for every word embedding is well below 90%. We therefore tuned a hyper parameter by setting ‘trainable’ to ‘True’ which results in updating the weights of word vectors in real-time. The vectors were uniformly distributed between the range of -0.5 and 0.5 for the tokens that were not found with word embedding.

**Table 9:** Statistics of matched words in each word embedding.

Word Embedding	Number of Matched Tokens	Percentage
Word2Vec (CBOW)	51850	79.8%
Word2Vec (Skip-gram)	51850	79.8%
GloVe	46351	71.4%
fastText	54377	83.7%

### 5.3 CNN layer

The application of CNN and its variations play an important part in Computer Vision and Natural Language Processing. Santos *et al*. [32] proposed a language-independent character-level feature extraction method, utilizing CNN for sequence labelling tasks. Applying this method, both Chiu *et al*. [33] and Ma *et al*. [34] achieved a good performance in POS tagging and NER. Karim *et al*. [29] used a variant of Densely Connected Network proposed by Lee *et al*. [47] for the first time in Bengali NER, using a large dataset, whereas Rifat *et al*. [28] used CNN on a small dataset to extract character level features.

Bangla words have a dynamic nature. The addition of a suffix or a prefix to the root of a word can change the meaning of the word, depending on the context of other words in a sequence. Table 10 illustrates how suffixes and prefixes alter the meaning of root words.

**Table 10:** Changing nature of the root words with the addition of suffixes and prefixes.

Entity	Root Word	Suffix	Prefix	Converted Word
Person	রাম	অঘা	-	অঘারাম
	জামাল	-	পুর	জামালপুর
	শুভ	অ	-	অশুভ
Location	পূর্বাচল	-	এ	পূর্বাচলে
Organization	বিদ্যালয়	-	এর	বিদ্যালয়ের
Quantity	এক	-	শ	একশ
	এক	-	টি	একটি
Currency	টাকা	-	র	টাকার
	টাকা	-	য়	টাকায়

**Fig 8:**
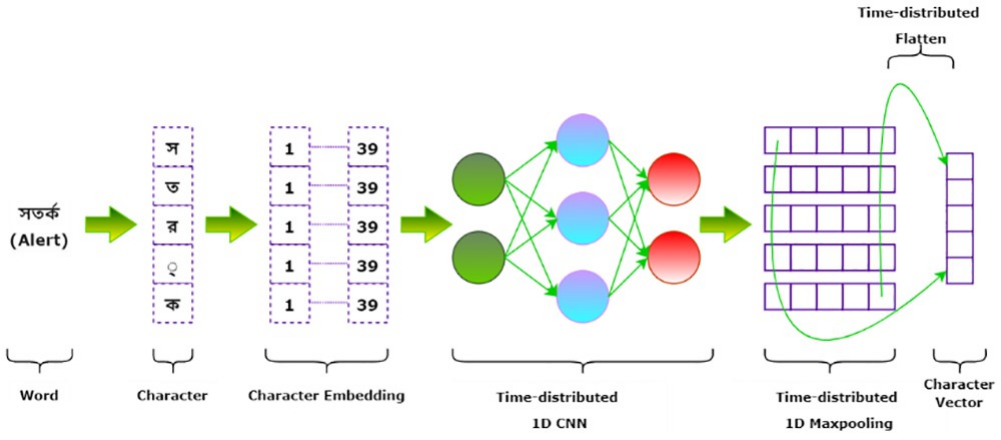
Character level feature extraction with CNN.

We have used CNN to extract character-level features to make our models understand the complex structure of Bangla words. For each word in a sentence, Fig 8 illustrates the character level feature extraction process utilizing Convolutional Neural Network (CNN). Initially, a character set is created which includes all the unique characters present in our dataset. There are 91 characters in this collection, including punctuation marks and special characters. Since our entire dataset includes 91 unique characters, the character input shape is (n, 91). We introduce character embedding of 38 dimensions and the vectors are uniformly distributed within the range of -0.5 and 0.5. There are three time-distributed layers to achieve character representations. A one-dimensional CNN layer with the activation function *tanh* which takes the character embeddings and follows a Maxpooling layer to capture the most relevant character features of the sentences. The outputs from the Maxpooling layer are fitted into a layer to convert the pooled feature map to a one-dimensional character vector.

### 5.4 Bidirectional LSTM and bidirectional GRU layer

Recurrent Neural Networks (RNN) have widely applied in Natural Language Processing. RNN incorporates a memory feature that enables the effect of prior inputs on future predictions. There are four sentences in Table 11. If we make predictions for Sentence 1, *ঢাকা (Dhaka)* should be predicted as *B-LOC*. The use of RNN in NER tasks helps to identify *Dhaka* as a location entity because RNN is good at handling short-term dependencies. If we also want to make predictions for Sentence 2, *ঢাকা (Dhaka)* should be predicted as *B-ORG* since *Dhaka College* belongs to an organization entity. In this sentence, it is necessary to learn long-term dependency to predict *Dhaka*. RNN is incapable of handling such long-term dependencies due to the vanishing gradient problem. To handle both long and short-term dependencies effectively, two variants of RNN have been developed: LSTM and GRU. LSTM [48] has three gates where the cell state acts like memory and transfers the information to the next part of the sequence. GRU is a simpler version of LSTM having only two gates. GRU uses hidden states rather than cell states to transfer the information to the next part of the sequence. However, feed-forward LSTM and GRU have some limitations. If we make predictions for Sentence 3 using feed-forward LSTM or GRU, the model will take the context of the preceding word *Apple* into account and correctly identify *আপেল (Apple)* as a Non-Named Entity. The problem with the feed-forward method arises when it is important to predict a word correctly considering the context of both preceding and future words. For instance, *Apple* in Sentence 4 belongs to person entity. As there are no words before *Apple* to understand that this belongs to a person entity, the feed-forward method cannot predict it correctly. To deal with this issue, we employed bidirectional LSTM and GRU in building the model. The bidirectional approach identifies a word in a predefined class by considering the context of the previous and following words in a sequence. The application of bidirectional LSTM has produced outstanding results in English, Spanish, German, and Dutch NER [33, 34, 49]. Young *et al*. [50] conducted a study of 190 research papers on various NLP tasks and found that researchers have not yet reached consensus on which variant of RNN is best for the NLP tasks. We have therefore used both bidirectional LSTM and GRU to compare their performance. All the models use 200 neurons in each direction (forward, backward) for both variants. The hidden states generated from forward and backward LSTM or GRU jointly produce the final hidden state.

**Table 11:** Example sequence for prediction.

**Sentence-1**	*ঢাকা* জেলার আয়তন ৩০৬ বর্গকিলোমিটার। (The area of *Dhaka* district is 306 square kilometres.)
**Sentence-2**	দেশের শীর্ষস্থানীয় ঐতিহ্যবাহী কলেজ এবং উপমহাদেশের প্রথম আধুনিক শিক্ষাপ্রতিষ্ঠান হিসেবে *ঢাকা কলেজ* প্রতিষ্ঠিত হয়। (*Dhaka College* was established as one of the top traditional colleges in the country and the first modern educational institution in the subcontinent.)
**Sentence-3**	তাদের বাগানে সারি সারি *আপেল* গাছ আছে। (There are rows and rows of *apple* trees in their garden.)
**Sentence-4**	*আপেল মাহমুদ* এই বাগানের রক্ষনাবেক্ষনের দায়িত্বে আছেন। (*Apple Mahmud* is in charge of the maintenance of this garden.)

### 5.5 CRF layer

Lafferty *et al*. [51] pioneered the use of CRF in building probabilistic models for sequence labeling tasks. In natural language processing, CRFs are used to predict a sequence, taking into account the context of the whole sequence.

Many state-of-the-art architectures have shown that the addition of a CRF layer after a BLSTM layer is essential for good NER performance (20,33,49,52). Lample *et al*. [49] and Akbik *et al*. [52] used a variant of the BLSTM-CRF model proposed by Huang *et al*. [53] for sequence labeling tasks. Ma *et al*. [34] enhanced the BLSTM-CNN model with a CRF layer for POS tagging and NER tasks.

In this research, we utilized Linear Chain CRF to model the full sequence of labels associated with a sequence of inputs. The final hidden states generated from the bidirectional LSTM or GRU are followed by a linear layer. The output produced by the linear layer is *X = {x*_*1*_, *x*_*2*_, *x*_*3*_,. . . ., *x*_*n*_*}*. The CRF layer takes all the outputs from the preceding linear layer as inputs and calculates the conditional log probability for the output sequence *y = {y*_*1*_, *y*_*2*_, *y*_*3*_,. . . ., *y*_*n*_*}* by Eq (2).

$$P\left(y|X\right)=\frac{\text{exp}\;\left(W\right)}{Z(X)}$$ (2)

*where*,

$$W=\sum_{n=1}^{N}E\left(x_{n}{,y}_{n}\right)+\sum_{n=1}^{N-1}T\left(y_{n},y_{n+1}\right)$$ (3)

and,

$$Z(X)=\sum_{y}\text{exp}\;\left(W\right)$$ (4)

There are two functions in Eq (3): Emission (E) and Transition (T). The emission score for the word at index *n* comes from the hidden state of the BLSTM or BGRU at time step *n*. The transition scores are stored in a matrix T. The probability of a sequence y given X is the exponential of a sum of two terms. One expresses the preferred value for each element in the sequence y, given the associated input, and the other is the sum of pairwise preferences between adjacent labels. Here, *Z(X)* denotes the normalization factor. According to probability theory, all conditional probabilities must sum up to 1. This is why the normalization factor *Z(X)* is included.

### 5.6 Model training

We have trained a total of *36* models. The batch size was set to *100*, the learning algorithm was *Nadam* with a learning rate of *0*.*001*, and training was conducted over *35* epochs for all experiments. Due to the large number of non-named entity samples in the dataset, bias occurs during training time. We have therefore utilized two types of loss functions considering the data imbalance: Focal Loss, and Dice Loss. The following five models were compiled with *sparse categorical focal loss*: BLSTM, BGRU, BLSTM+CNN+Dropout, BLSTM+CNN-Dropout, BGRU+CNN. *Focal Loss* deals with the class imbalance problem by penalizing hard to classify samples. The remaining models were compiled with *dice loss*. *Dice Loss* also handles training bias for a highly imbalanced dataset. We have applied the loss functions mentioned above provided by the *Python Package Index* which are compatible with Tensorflow 2.0.1 and higher versions.

### 5.7 Model evaluation

The most typical approach for evaluating NER tasks is to measure Precision, Recall, and F1 scores at the token level. However, it is more efficient to evaluate with metrics at the named entity level. We evaluated all the experimental models in accordance with SemEval’13. The evaluation procedure of SemEval’13 considers the following scenarios on top of the MUC defined metrics:

1. COR (Correct): If the predicted label and the gold standard annotation are same.
2. INC (Incorrect): If there is a mismatch between the predicted label category for a chunk and the gold standard annotation.
3. PAR (Partial): If the predicted label for a chunk partially matches the gold standard annotation.
4. SPU (Spurious): If the model labels an entity that does not exist in the gold standard annotation.
5. MIS (Missing): If a chunk is not captured by a model.

Table 12 shows the evaluation procedure for a sample sentence with respect to the gold standard annotation and the predicted labels. Both the boundaries and the type are quantified using two metrics: POS and ACT.

**Table 12:** Sample evaluation.

Sentence	Gold Standard Annotation	Predicted Label	Evaluation
চীনের	B-LOC	B-LOC	***COR***
ছয়	B-QTY	B-QTY	***COR***
অশ্বশক্তির	I-QTY	I-QTY	
একটি	B-QTY	B-PCT	***INC***
শ্যালো	O	B-PER	***SPU***
যন্ত্রের	O	O	
দাম	O	O	
১৪	B-CUR	B-CUR	***PAR***
হাজার	I-CUR	I-CUR	
টাকার	I-CUR	O	
আশপাশে	O	O	
।	O	O	


$$\begin{array}{cc} POS\;\left(Possible\right) & =COR+INC+PAR+MIS\\ & =TP+FN \end{array}$$

$$\begin{array}{cc} ACT\;\left(Actual\right) & =COR+INC+PAR+SPU\\ & =TP+FP \end{array}$$

*POS* denotes the number of gold-standard annotations that contribute to the final score and *ACT* denotes the number of annotations generated by a model. Evaluation results are reported using standard Precision, Recall, and F1 metrics where *Precision (P)* is the percentage of correctly named entities found by a model, *Recall (R)* is the percentage of named entities in our NER corpus that are found by a model, and *F1* score is the harmonic mean of *Precision* and *Recall*. Precision, Recall and F1 can be calculated by the following equations for exact and partial matches.


$$P_{\left(Exact\right)}=\frac{COR}{ACT}=\frac{TP}{TP+FP}$$

$$P_{\left(Partial\right)}=\frac{COR\;+\;0.5\;*\;PAR}{ACT}$$

$$R_{\left(Exact\right)}=\frac{COR}{POS}=\frac{TP}{TP+FN}$$

$$R_{\left(Partial\right)}=\frac{COR\;+\;0.5\;*\;PAR}{POS}$$

$$F1=2*\frac{Preicision*Macro}{Preicision+Recal}$$

Precision, Recall, F1 were calculated for each type of entity considering both exact and partial matches. Subsequently their macro average measures were found. Calculating these metrics for each entity type allows evaluation of the level of difficulty of recognizing each entity type. These metrics were also calculated for all entities. The final score is the micro averaged F1 measure, which is calculated without distinction over all entity types. The main advantage of the micro F1 is that it takes into account all possible types of errors which can be made by the model.

## 6. Experiments and result analysis

All models were first trained with 8,04,637 tokens and then tested with 2,01,154 tokens. We performed some experiments to evaluate the performance of different word embeddings, CNN layer integration, CRF layer integration, BLSTM and BGRU in producing sequentially and contextually meaningful sentences, as well as the effect of dropout. Tables 13–15 include both macro averaged exact and partial Precision, Recall, and F1 scores respectively, and Table 16 includes micro averaged F1 scores for all the models. We describe and analyse the findings of each experiment in the following subsections.

**Table 13:** Exact and partial precision scores.

Model	Feature	Partial Precision (%)				Exact Precision (%)			
		w2v cbow	w2v skipgram	glove	fastText cbow	w2v cbow	w2v skipgram	glove	fastText cbow
BLSTM+CNN+Dropout	W+C	94.55	93.46	92.95	92.98	86.54	88.07	88.15	84.94
BLSTM+CNN-Dropout	W+C	93.48	92.16	94.10	92.87	88.15	85.48	86.81	84.32
BGRU+CNN	W+C	93.42	92.35	92.47	92.47	87.49	87.41	86.03	86.03
BLSTM+CNN+CRF	W+C	93.11	91.65	91.75	93.71	88.98	84.65	86.66	89.44
BGRU+CNN+CRF	W+C	93.01	93.81	93.62	92.76	88.85	90.34	89.49	89.08
BLSTM	W	89.56	90.87	91.51	88.42	81.00	83.90	83.00	76.36
BGRU	W	89.44	90.22	90.91	87.11	82.87	83.37	82.72	78.71
BLSTM+CRF	W	90.30	92.99	90.30	90.67	85.46	88.71	84.71	80.36
BGRU+CRF	W	91.84	91.45	91.84	91.88	86.20	86.36	85.92	87.71

**Table 14:** Exact and partial recall scores.

Model	Feature	Partial Recall (%)				Exact Recall (%)			
		w2v cbow	w2v skipgram	glove	fastText cbow	w2v cbow	w2v skipgram	glove	fastText cbow
BLSTM+CNN+Dropout	W+C	79.31	82.22	84.31	74.68	68.52	73.46	72.76	61.88
BLSTM+CNN-Dropout	W+C	78.65	85.80	86.43	77.38	70.80	76.79	77.00	65.03
BGRU+CNN	W+C	86.12	90.19	88.89	88.89	77.35	81.05	79.62	79.62
BLSTM+CNN+CRF	W+C	90.90	81.22	90.24	83.80	84.14	74.72	82.16	76.14
BGRU+CNN+CRF	W+C	91.61	90.20	91.09	85.73	86.19	83.59	84.53	77.94
BLSTM	W	79.03	81.78	84.73	68.18	70.60	70.90	73.00	51.56
BGRU	W	86.31	86.36	85.61	77.67	75.87	75.28	73.42	62.14
BLSTM+CRF	W	86.34	84.24	86.34	78.07	80.97	76.58	75.95	63.14
BGRU+CRF	W	86.83	84.31	86.83	79.87	79.09	79.05	77.34	69.51

**Table 15:** Exact and partial F1 scores.

Model	Feature	Partial F1 (%)				Exact F1 (%)			
		w2v cbow	w2v skipgram	glove	fastText cbow	w2v cbow	w2v skipgram	glove	fastText cbow
BLSTM+CNN+Dropout	W+C	85.31	86.88	88.27	81.09	76.48	80.10	79.72	71.60
BLSTM+CNN-Dropout	W+C	84.33	88.64	89.24	82.39	78.53	80.90	81.61	73.43
BGRU+CNN	W+C	89.36	91.15	90.52	90.52	82.11	84.11	82.70	82.70
BLSTM+CNN+CRF	W+C	91.93	85.00	90.92	87.77	86.49	79.38	84.35	82.26
BGRU+CNN+CRF	W+C	92.31	91.90	92.31	88.89	87.50	86.83	86.94	83.14
BLSTM	W	83.43	85.73	87.76	74.41	75.40	76.90	77.60	61.56
BGRU	W	87.73	88.10	88.09	80.95	79.22	79.12	77.79	69.45
BLSTM+CRF	W	88.43	88.16	88.43	83.63	83.16	82.20	80.09	70.72
BGRU+CRF	W	88.84	89.25	88.84	84.70	82.49	82.54	81.40	77.56

**Table 16:** Micro F1 scores.

Model	Feature	Micro F1 (%)			
		w2v cbow	w2v skipgram	glove	fastText cbow
BLSTM+CNN+Dropout	W+C	96.95	97.36	97.34	96.30
BLSTM+CNN-Dropout	W+C	97.08	97.56	97.64	96.48
BGRU+CNN	W+C	97.66	97.94	97.74	97.74
BLSTM+CNN+CRF	W+C	98.15	97.07	97.88	97.44
BGRU+CNN+CRF	W+C	98.32	98.21	98.23	97.64
BLSTM	W	96.79	97.04	97.14	95.12
BGRU	W	97.28	97.26	97.15	95.97
BLSTM+CRF	W	97.29	97.58	97.29	95.96
BGRU+CRF	W	97.47	97.64	97.47	96.83

### 6.1 Hybrid VS Non-hybrid model

Our experimental set of nine models includes both hybrid and non-hybrid models. We have made variations of two non-hybrid models to enhance performance. Table 17 shows the performance improvement of hybrid models compared to non-hybrids. Since the F1 score provides combined information about precision and recall of a model, future analysis will be based on the F1 score only. We have compared the models BLSTM and BGRU with their variations, taking the highest values from each row in Tables 15 and 16 where the partial, exact, and micro F1 scores of each model are listed. In Table 17, we have denoted the non-hybrid models as the numerator and the corresponding hybrid models with those that will be compared as the denominator to find out the ratio of the F1 scores. Observing the column *Improved Performance*, it is clear that non-hybrid models perform better than hybrid models. BLSTM+CNN+CRF and BGRU+CNN+CRF outperform their respective BLSTM and BGRU variations.

**Table 17:** Comparison of hybrid and non-hybrid models’ F1 scores.

Evaluation	Numerator	Max F1 (Numerator)	Denominator	Max F1 (Denominator)	Ratio (%)	Improved Performance (%)
Partial	BLSTM	87.76	BLSTM+CRF	88.43	99.24	0.76
			BLSTM+CNN+CRF	91.93	95.46	4.54
			BLSTM+CNN-Dropout	89.24	98.34	1.66
			BLSTM+CNN+Dropout	88.27	99.42	0.58
Exact	BLSTM	77.6	BLSTM+CRF	83.16	93.31	6.69
			BLSTM+CNN+CRF	86.49	89.72	10.28
			BLSTM+CNN-Dropout	81.61	95.08	4.92
			BLSTM+CNN+Dropout	80.1	96.87	3.13
Micro	BLSTM	95.12	BLSTM+CRF	97.58	97.47	2.53
			BLSTM+CNN+CRF	98.15	96.91	3.09
			BLSTM+CNN-Dropout	97.64	97.41	2.59
			BLSTM+CNN+Dropout	97.36	97.69	2.31
Partial	BGRU	88.1	BGRU+CRF	89.25	98.71	1.29
			BGRU+CNN	91.15	96.65	3.35
			BGRU+CNN+CRF	92.31	95.43	4.57
Exact	BGRU	79.22	BGRU+CRF	82.54	95.97	4.03
			BGRU+CNN	84.11	94.18	5.82
			BGRU+CNN+CRF	87.50	90.53	9.47
Micro	BGRU	97.28	BGRU+CRF	97.64	99.63	0.37
			BGRU+CNN	97.94	99.32	0.68
			BGRU+CNN+CRF	98.32	98.94	1.06

### 6.2 Effects of different word embeddings

We experimented with word2Vec (cbow), word2vec (skip-gram), glove, fasttext (cbow) in order to compare the effects of different word embeddings. Table 18 includes the maximum and minimum scores for precision, recall, and F1 obtained for each word embedding. We have taken the highest and lowest values from each column in Tables 13–16 where the precision, recall, and F1 scores of each model for each word embedding are listed. We have compared the values of Table 18 along each row to get the row-wise highest (green shaded) and lowest values (cyan shaded). It is obvious that fasttext results in the worst scores for all evaluation criteria. The remaining three word embeddings perform much better. There are six green shaded values for word2vec (cbow), and one for word2vec (skip-gram) and glove. Both word2vec (cbow) and glove achieved the best F1 score of 92.31% for the partial match. In case of exact match, word2vec (cbow) aids in obtaining the best F1 score of 87.50% which is 0.56% higher than the score reported for glove. For micro averaged F1, word2vec (cbow) provides the best F1 score of 98.32% which is 0.09% better than the score obtained by using glove. From the analysis of macro F1 scores, we can conclude that word2vec (cbow) outperforms all other word embeddings.

**Table 18:** Comparison of results between four word embeddings.

Evaluation Metrics			w2v cbow	w2v skip-gram	glove	fastText
Precision	Partial	Max	94.55	93.81	94.10	93.71
		Min	89.44	90.22	90.30	87.11
	Exact	Max	88.98	90.34	89.49	89.44
		Min	81.00	83.37	82.27	76.36
Recall	Partial	Max	91.61	90.20	91.09	88.89
		Min	78.65	81.22	84.31	68.18
	Exact	Max	86.19	83.59	84.53	79.62
		Min	68.52	70.90	72.76	51.56
F1	Partial	Max	92.31	91.90	92.31	90.52
		Min	83.43	85.00	87.76	74.41
	Exact	Max	87.50	86.83	86.94	83.14
		Min	75.40	76.90	77.60	61.56
	Micro	Max	98.32	98.21	98.23	97.74
		Min	96.79	97.04	97.14	95.12

### 6.3 CNN layer integration

As previously stated, the nine basic models belong to two categories depending on their integration with character-level features. For character level feature extraction, we experimented with CNN. In Tables 13–16, the term ‘W+C’ refers to a model that uses word embedding along with the CNN extracted character level features, and ‘W’ refers to a model that uses only word embedding. Table 19 portrays significant findings. We have taken the highest and lowest precision, recall, and F1 scores for both types of models from Tables 13–16. We highlight the highest and lowest precision, recall, and F1 scores gained for type-1 models with those of type-2 models. We highlight the best scores with green shades and the worst scores with cyan shades for each evaluation criteria. It can be seen from Table 19 that the best scores for precision, recall, and F1 were gained with type-1 models. It is also clear that the worst scores for precision, recall, and F1 come from type-2 models. Integration of a CNN layer into a type-1 model improves macro F1 scores by 3.06% (partial), 4.34% (exact), and micro F1 scores by 0.68%. Comparing the results of these two types of models, we can conclude that the addition of character level morphological features can boost the performance for Bengali NER.

**Table 19:** Comparison of results on integrating CNN layer.

Type	Feature	Evaluation		Precision	Recall	F1
Type-1	W+C	Partial	Max	94.55	91.61	92.31
			Min	91.65	74.68	81.09
		Exact	Max	90.34	86.19	87.50
			Min	84.32	61.88	70.60
		Micro	Max			98.32
			Min			96.30
Type-2	W	Partial	Max	91.88	86.83	89.25
			Min	87.11	68.18	74.41
		Exact	Max	87.71	80.97	83.16
			Min	76.36	51.56	61.56
		Micro	Max			97.64
			Min			95.12

### 6.4 CRF layer integration

We have made some variations to the output layer for experimental purposes. Four of the nine basic models use CRF as the output probability and the other five use softmax. Table 20 shows the performance measures for CRF based and non-CRF based models. The highest and lowest precision, recall, and F1 scores for both types of models can be seen in Tables 13–16. We have compared the highest and lowest precision, recall, and F1 scores gained for CRF based models with those of non-CRF based models, highlighting the best scores with green shade and the worst scores with cyan shade for each evaluation criteria. It can be observed from Table 20 that six of the best scores are achieved with CRF based models and only one with a non-CRF based model. It is worth noting that both the best and worst precision scores for partial matches along with six of the worst scores for other evaluation measures, come from Non-CRF based models. Integration of the CRF layer improves macro F1 scores by 1.16% (partial), 1.01% (exact), and micro F1 scores by 0.38%. Comparing the results for two output probabilities, we can conclude that CRF performs better in predicting the tag sequence for Bengali NER.

**Table 20:** Comparison of results on integrating CRF layer.

Output Probability	Evaluation		Precision	Recall	F1
CRF	Partial	Max	93.81	91.61	92.31
		Min	90.30	78.07	83.63
	Exact	Max	90.34	86.19	87.50
		Min	80.36	63.14	70.60
	Micro	Max			98.32
		Min			95.96
Softmax	Partial	Max	94.55	90.19	91.15
		Min	87.11	68.18	74.41
	Exact	Max	88.15	81.05	86.49
		Min	76.36	51.56	61.56
	Micro	Max			97.94
		Min			95.12

### 6.5 BGRU VS BLSTM

We have experimented with Bidirectional GRU and LSTM to model sequential information for Bengali NER. Four of the nine basic models apply BGRU and the other five apply BLSTM. Table 21 shows the performance measures for BGRU based and BLSTM based models. We denote BGRU and BLSTM based models as Group-1 and Group-2 respectively. We have taken the highest and lowest precision, recall, and F1 scores for both types of models from Tables 13–16. We have compared the highest and lowest precision, recall, and F1 scores for Group-1 models with those for Group-2 models, highlighting the best scores with green shade and the worst scores with cyan shade for each evaluation criteria. From Table 21, it can be observed that six of the best scores result from Group-1 models and only one from Group-2. It is worth noting that both the best and worst precision scores for partial matches as well as six of the worst scores for other evaluation measures, come from Group-2. Utilization of BGRU improves macro F1 scores by 0.38% (partial), 3.15% (exact), and micro F1 scores by 0.17%. GRU is more efficient in terms of computing and memory use than LSTM. Moreover, GRU is less complicated since it utilizes just two gates: Reset and Update. The reset gate adds new information to the previous output and the update gate specifies how much information from the previous output should flow on for the current computation. Comparing the results for the two variants of RNN, we can conclude that BGRU based models perform better in analyzing the context of sequences for Bengali NER.

**Table 21:** Comparison of results between BGRU and BLSTM based models.

Group	Evaluation		Precision	Recall	F1
Group-1 (BGRU)	Partial	Max	93.81	91.61	92.31
		Min	87.11	77.67	80.95
	Exact	Max	90.34	86.19	87.50
		Min	78.71	62.14	69.45
	Micro	Max			98.32
		Min			95.97
Group-2 (BLSTM)	Partial	Max	94.55	90.90	91.93
		Min	88.42	68.18	74.41
	Exact	Max	89.44	84.14	84.35
		Min	76.36	51.56	61.56
	Micro	Max			98.15
		Min			95.12

### 6.6 Dropout effect

**Fig 9:**
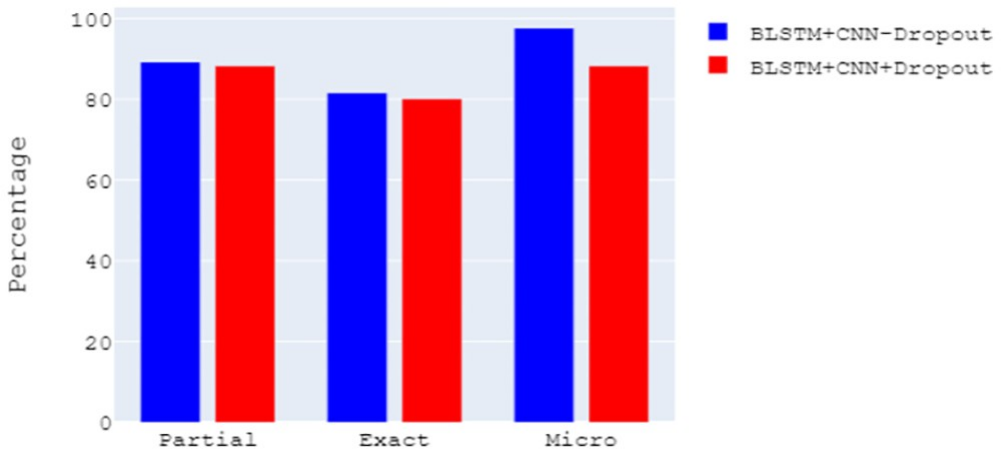
Dropout effect.

Dropout concept was introduced by Srivastava *et al*. [54] to prevent neural networks from overfitting. This research [54] demonstrated that dropout improved neural network performance and improved results on many benchmark datasets. We randomly dropped units from the BLSTM and BGRU layers for experimental purposes. Fig 9 depicts the F1 scores of two models before and after adding the dropout. To indicate the use of dropout in a model, ‘+Dropout’ has been used in the name of the model. We have taken the highest F1 scores of two models under four word embeddings from Table 15. It is clear that dropout has a negative effect on performance. Not including dropout improves macro F1 scores by 0.97% (partial), 1.51% (exact), and micro F1 scores by 0.28%. As an example, the dropout effect on a single model is shown. Dropout had a negative effect on the results of other models as well. One possible reason is that all the words contribute to understanding the syntactic and semantic meaning of a sequence and help to predict the named entity chunk. The network is forced to generalize through dropouts. In our case, the learning capacity required for the target dataset is more than the current capacity.

### 6.7 Complementary behavior analysis

We have selected 9 models from Table 15 in order to analyze the complementary behavior of the models against Named Entity (NE) classes based on their exact F1 scores. The word embeddings [55] for the models that performed best are shown in Table 22. The actual count of each NE class in the test set and the number of NE classes that the models correctly classified are also included in Table 22. The maximum count of accurately classified NE classes is shown by the yellow shaded scores. It is noteworthy from Table 22 that the model BGRU+CNN+CRF has produced the maximum number of correct predictions for all NE classes. Both the BLSTM+CNN+CRF and the BGRU+CNN+CRF were found to perform equally well for the class *B-PCT*. Eight models have performed closely to the model BGRU+CNN+CRF for the classes *I-QTY*, *B-PCT*, *I-PCT*, *B-CUR*, and *I-CUR*. Additionally, Table 22 also reflects the final outcomes of the experiments in subsections 6.1–6.6. In order to conduct a more thorough investigation of the complimentary behavior analysis, we combined all the models and used the majority voting approach on the predictions. Table 10 shows that the results of ensemble learning have outperformed some models’ individual performances except the model BGRU+CNN+CRF.

**Table 22:** Count of correctly predicted NE classes.

**I-CUR**	**692**		676	682	687	679	676		682	684	686	689		**574**
**B-CUR**	**342**		314	315	328	321	317		317	321	328	329		**285**
**I-PCT**	**348**		326	329	333	333	335		334	330	335	337		**293**
**B-PCT**	**253**		222	223	232	227	225		225	227	230	232		**215**
**I-QTY**	**1,096**		991	1019	1046	1029	1026		1019	1030	1037	1048		**1015**
**B-QTY**	**2,416**		1924	1969	2177	2009	1969		1966	1990	2032	2208		**2065**
**I-ORG**	**2,212**		1490	1873	1966	1648	1768		1871	1883	1971	1973		**1786**
**B-OGR**	**2,328**		1363	1696	1814	1486	1401		1634	1800	1833	1861		**1690**
**I-LOC**	**406**		182	283	306	259	240		253	285	303	307		**276**
**B-LOC**	**4,723**		3771	4097	4219	3988	3925		4030	4125	4215	4354		**4145**
**I-PER**	**3,344**		2997	3009	3211	3147	3168		3056	3085	3210	3213		**3090**
**B-PER**	**4,356**		3388	3519	3729	3583	3500		3457	3578	3674	3806		**3648**
**Word Embedding**	-		glove	word2vec (cbow)	word2vec (cbow)	glove	word2vec (skipgram)		word2vec (cbow)	word2vec (skipgram)	word2vec (skipgram)	word2vec (skipgram)		
**Model**	Actual Count		BLSTM	BLSTM + CRF	BLSTM + CNN + CRF	BLSTM + CNN − Dropout	BLSTM + CNN + Dropout		BGRU	BGRU + CRF	BGRU + CNN	BGRU + CNN + CRF		**Combined**

### 6.8 Qualitative analysis on sentence-level named entity extraction

We have conducted statistical assessments of our models in sections 6.1–6.7. In this section, we evaluate the consistency of quantitative analysis regarding the models’ ability to extract named entities from challenging test samples.

A sentence from the test set is shown in Table 23 along with its real tag and the predicted tag from the BLSTM-based models. The non-hybrid model BLSTM and the hybrid model BLSTM+CNN-Dropout have a higher number of incorrect predictions for this sample sentence. The BLSTM+CNN+CRF model classifies all the tokens correctly. Other models classify tokens 13, 16, and 18 incorrectly. Token 13 actually belongs to the *location* entity, while models except BLSTM+CNN+CRF classify it as *other* and *organization*. Although token 16 belongs to the *person* entity, it is classified as other in both the BLSTM+CNN-Dropout and BLSTM models. Only the model BLSTM+CRF misclassifies token 18. It is important to note that although token 18 is sometimes used as a person’s name, we have tagged it as *other* given the context of the sentence. The Table 23 illustrates that incorporating CRF into the base model BLSTM (BLSTM+CRF) enables accurate prediction of tokens 13 and 16, while it falls short in predicting token 18. When character-level embedding is added to the base model BLSTM without using dropout, it (BLSTM+CNN) successfully predicts token 18 but fails to accurately predict token 13. In contrast, the addition of CRF with the hybrid model BLSTM+CNN (BLSTM+CNN+CRF) achieves correct predictions for all tokens.

**Table 23:** Test sample-1.

1	Sentence	Actual Tag	BLSTM + CNN + CRF	BLSTM + CNN + Dropout	BLSTM + CNN − Dropout	BLSTM + CRF	BLSTM
2	সে	O	O	O	O	O	O
3	সময়ের	O	O	O	O	O	O
4	পররাষ্ট্রসচিব	O	O	O	O	O	O
5	ফখরুদ্দিন	B-PER	B-PER	B-PER	B-PER	B-PER	B-PER
6	আহমেদ	I-PER	I-PER	I-PER	I-PER	I-PER	I-PER
7	১৫	O	O	O	O	O	O
8	আগস্টের	O	O	O	O	O	O
9	পূর্ববর্তী	O	O	O	O	O	O
10	অবস্থাকে	O	O	O	O	O	O
11	১৯৬৫	O	O	O	O	O	O
12	সালে	O	O	O	O	O	O
13	ঘানার	B-LOC	B-LOC	O	O	B-LOC	B-ORG
14	জনপ্রিয়	O	O	O	O	O	O
15	রাষ্ট্রনায়ক	O	O	O	O	O	O
16	নক্রুমার	B-PER	B-PER	B-PER	O	B-PER	O
17	বিরুদ্ধে	O	O	O	O	O	O
18	তরুণ	O	O	O	O	B-PER	O
19	অফিসারদের	O	O	O	O	O	O
20	অভ্যুত্থান-পূর্ব	O	O	O	O	O	O
21	অবস্থার	O	O	O	O	O	O
22	সঙ্গে	O	O	O	O	O	O
23	তুলনা	O	O	O	O	O	O
24	করেছেন	O	O	O	O	O	O
25	।	O	O	O	O	O	O

The second test sample is shown in Table 24 along with its real tag and the predicted tag from the BGRU-based models. The non-hybrid model BGRU has a higher number of incorrect predictions for this sample sentence, resulting in four misclassifications. The BGRU+CNN+CRF model classifies all the tokens correctly. Other models misclassify tokens 2, 3, 12, and 13. Token 2 actually belongs to the *location* entity, while both the BGRU+CRF and BGRU models classify it as a *person* entity. The BGRU+CRF and BGRU models differ in that the BGRU model classifies tokens 2, 3, 4, and 5 as chunks of a *person* entity. Similar occurrences have been found for the tokens 12 and 13 by the BGRU+CNN and BGRU models. Table 24 demonstrates that by integrating CRF into the base model BGRU (BGRU+CRF), the model is able to correctly identify token 3 as the start of a chunk. Furthermore, it accurately predicts tokens 12 and 13, although it struggles to classify token 2 as a *location* entity. The model poses a greater challenge in correctly classifying token 12, which stands for the rank name of a specific profession. The synergy achieved by combining word embedding and character-level embedding with the base model BGRU outperforms alternative approaches for classifying all token correctly.

**Table 24:** Test sample-2.

1	Sentence	Actual Tag	BGRU + CNN + CRF	BGRU + CNN	BGRU + CRF	BGRU
2	লাহোরে	B-LOC	B-LOC	B-LOC	B-PER	B-PER
3	মাহমুদ	B-PER	B-PER	B-PER	B-PER	I-PER
4	আলী	I-PER	I-PER	I-PER	I-PER	I-PER
5	কাসুরির	I-PER	I-PER	I-PER	I-PER	I-PER
6	বাসায়	O	O	O	O	O
7	প্রেসিডেন্ট	O	O	O	O	O
8	আইয়ুব	B-PER	B-PER	B-PER	B-PER	B-PER
9	খানের	I-PER	I-PER	I-PER	I-PER	I-PER
10	সামরিক	O	O	O	O	O
11	সচিব	O	O	O	O	O
12	ব্রিগেডিয়ার	O	O	B-PER	O	B-PER
13	পীরজাদার	B-PER	B-PER	B-PER	B-PER	I-PER
14	সঙ্গে	O	O	O	O	O
15	ন্যাপের	B-ORG	B-ORG	B-ORG	B-ORG	B-ORG
16	কেন্দ্রীয়	O	O	O	O	O
17	কমিটির	O	O	O	O	O
18	সদস্য	O	O	O	O	O
19	মহিউদ্দিন	B-PER	B-PER	B-PER	B-PER	B-PER
20	আহমেদ	I-PER	I-PER	I-PER	I-PER	I-PER
21	ও	O	O	O	O	O
22	আহমেদুল	B-PER	B-PER	B-PER	B-PER	B-PER
23	কবীরের	I-PER	I-PER	I-PER	I-PER	I-PER
24	গোপন	O	O	O	O	O
25	বৈঠক	O	O	O	O	O
26	হয়	O	O	O	O	O
27	।	O	O	O	O	O

### 6.9 Best model selection

**Fig 10:**
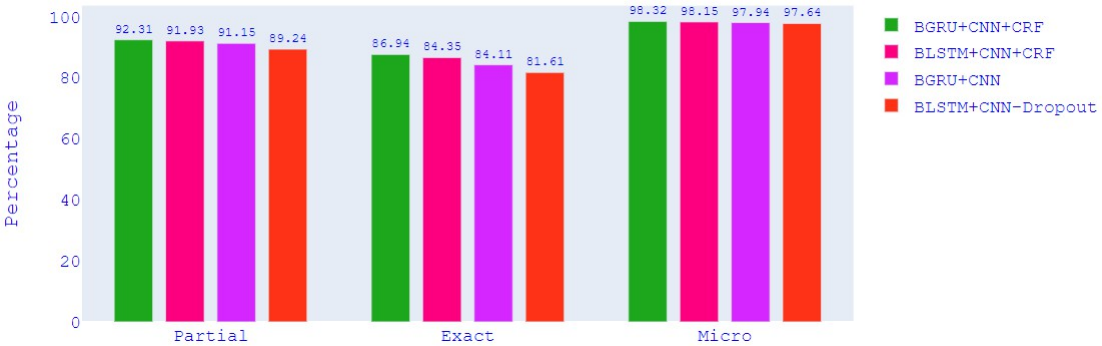
Comparison of results between four models.

The rigorous quantitative analysis conducted in sections 6.1–6.7 and the insightful qualitative analysis in section 6.8 strongly establish that integrating CNN for character-level feature extraction consistently results in improved accuracy. This significant finding influenced our initial decision to consider five models incorporating CNN in our research. Considering the detrimental impact of dropout on model performance as demonstrated in sections 6.6 and 6.8, a comparison was made among the four models that do not employ dropout. Fig 10 shows the macro and micro F1 scores of these four models. Sections 6.3, 6.4, and 6.8 reveal that the combination of BLSTM and character-level feature extraction has remarkable performance, further enhanced by CRF. Based on these findings, we eliminated the non-CRF models in the next phase, acknowledging the outstanding capabilities of the CRF-integrated approach. Through our comprehensive quantitative and qualitative analysis, it was observed that among the two remaining models (BLSTM+CNN+CRF and BGRU+CNN+CRF), the model BGRU+CNN+CRF has the most promising performance in Bengali Named Entity Recognition. The F1 scores for the model BGRU+CNN+CRF are shown in Table 25, along with the word embedding for which these values were obtained. It is noticeable that the glove and word2vec (cbow) improve performance. It can be concluded that BGRU+CNN+CRF provides the best performance using word2vec (cbow) word embedding.

**Table 25:** Macro and micro F1 scores of the best model.

Best Model	Evaluation	F1	Word Embedding
BGRU+CNN+CRF	Partial	92.31	w2v(cbow), glove
	Exact	87.50	w2v(cbow)
	Micro	92.31	w2v(cbow)

**Fig 11:**
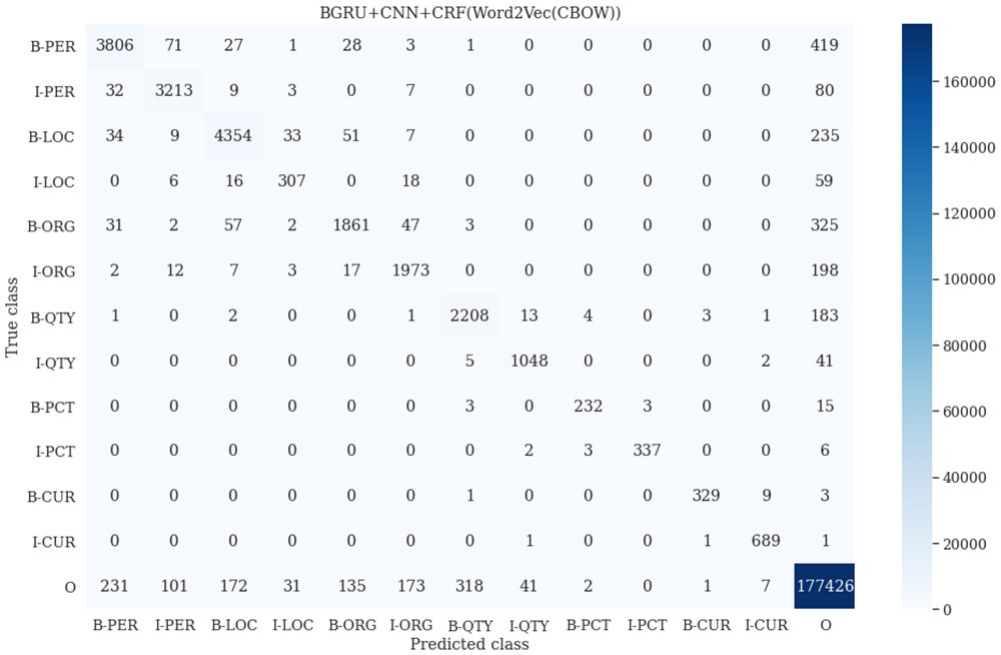
The graphical representation of confusion matrix of the best performing model.

The Confusion Matrix [55] in Fig 11 shows the results. The first column represents the correct classification for the token, and the first row shows the prediction produced by our proposed model for the token. The numbers on the main diagonal are tokens that are correctly predicted. The majority of tokens belong to the non-named entity class. Our best model correctly predicted 177,426 out of 178,638 non-named entity tokens. Fig 11 also shows that the majority of incorrect predictions occurred for ‘O’ and ‘B-ORG’. It is worth noting that there are similarities in the expressions of Quantity, Percentage, and Currency entities in Bengali. Moreover, the model was trained with a relatively small amount of data for these entity types. Nevertheless, our proposed model is capable of predicting these entities well.

The challenges introduced in Section I were evaluated for the best model to observe its robustness. Table 26 includes the gold-standard annotation along with the results of challenging sequences for word2vec (cbow) based BGRU+CNN+CRF. The predictions of the blue highlighted tokens are highlighted using green and red, indicating correct and incorrect predictions respectively. There are 29 target tokens in 18 sentences. Our model correctly predicted 25 tokens and made mistakes on 4 tokens. In sentence 5, রাবণের চিতায় (unquenchable fire) is an idiom. Our model predicts রাবণের as Person entity which is wrong. The token refers to Other in sentence 5 and to Person in sentence 6. We investigated the reason for this mistake and found that idioms are rarely used in writing. As a result, it was not possible to train the models with enough idiomatical sentences. In sentence 9, আড়াইহাজার (Araihazar) represents a Location while our model predicts আড়াইহাজার as Quantity. In sentence 11, এক (a) belongs to no named entity. Our model incorrectly predicts এক as Quantity. In sentence 16, the chunk শতকরা ৭৪.৭০ ভাগ (74.70%) belongs to the Percentage entity. Our model predicted শতকরা ৭৪.৭০ correctly but failed to predict ভাগ.

**Table 26:** Results on different challenges for BGRU+CNN+CRF (word2vec(cbow)).

Challenge	Sentence	Golden Annotation	Predicted Label
Multiple Meaning	1. মেয়েটির নাম বকুল (The girl’s name is Bakul) 2: বকুল ফুলের সুবাস বেশ মিষ্টি (The fragrance of the Medlar is so sweet)	1. O, O, B-PER 2. O, O, O, O, O	1. O, O, B-PER 2. O, O, O, O, O
	3. ঢাকা বাংলাদেশের রাজধানী (Dhaka is the capital of Bangladesh) 4. পাত্রটি ঢাকনা দিয়ে ঢাকা আছে (The pot is covered with a lid)	3. B-LOC, B-LOC, O 4. O, O, O, O, O	3. B-LOC, B-LOC, O 4. O, O, O, O, O
Idioms	5. আমি রাবণের চিতায় জ্বলছি (I am burning in unquenchable fire) 6. আমিই সেই রাবণ (I am the Ravana)	5. O, O, O, O 6. O, O, B-PER	5. O, B-PER, O, O 6. O, O, B-PER
Entity Inflection	7. তার বাড়ি কুড়িগ্রাম (His home is in Kurigram) 8. লেবুর হালি কুড়ি টাকা (Four lemon cost twenty taka)	7. O, O, B-LOC 8. O, O, B-CUR, I-CUR	7. O, O, B-LOC 8. O, O, B-CUR, I-CUR
	9. গ্রামটির নাম আড়াইহাজার (The name of the village is Araihazar) 10. জামাটির মূল্য আড়াই হাজার টাকা (The price of the dress is 2500 taka)	9. O, O, B-LOC 10. O, O, B-CUR, I-CUR, I-CUR	9. O, O, B-QTY 10. O, O, B-CUR, I-CUR, I-CUR
	11. সে এক বিশাল আয়োজন (It was a grand arrangement) 12. তার দুই ছেলে, এক মেয়ে (He has two sons and a daughter)	11. O, O, O, O 12. O, B-QTY, O, B-QTY, O	11. O, B-QTY, O, O 12. O, B-QTY, O, B-QTY, O
Multiple Expression	13. কাফি ব্যাংকে এক লাখ টাকা জমা রেখেছে (Kafy has deposited 1 lakh taka in the bank) 14. কাফি ব্যাংকে ১ লক্ষ টাকা জমা রেখেছে (Kafy has deposited 1 lakh taka in the bank)	13. B-PER, O, B-CUR, I-CUR, I-CUR, O, O 14. B-PER, O, B-CUR, I-CUR, I-CUR, O, O	13. B-PER, O, B-CUR, I-CUR, I-CUR, O, O 14. B-PER, O, B-CUR, I-CUR, I-CUR, O, O
	15. বাংলাদেশে স্বাক্ষরতার হার ৭৪.৭০% (The literacy rate in Bangladesh is 74.60%) 16. বাংলাদেশে স্বাক্ষরতার হার শতকরা ৭৪.৭০ ভাগ (The literacy rate in Bangladesh is 74.60%)	15. B-LOC, O, O, B-PCT 16. B-LOC, O, O,B-PCT, I-PCT,I-PCT	15. B-LOC, O, O, B-PCT 16. B-LOC, O, O, B-PCT, I-PCT, O
Expression Similarity	17. সে ১৭৫৭ টাকা দিয়ে নকশী কাঁথাটি কিনেছে (She has bought the nakshi kantha for 1757 taka) 18. ১৭৫৭ সালে পলাশীর যুদ্ধ হয় (The battle of Palashi took place in 1757)	17. O, B-CUR, I-CUR, O, O, O, O 18. O, O, O, O	17. O, B-CUR, I-CUR, O, O, O, O 18. O, O, O, O, O

We assessed these sentences using the word2vec (cbow) based BLSTM+CNN+CRF to gain a deeper understanding of the differences in performance with the model BGRU+CNN+CRF. We have observed that the misclassifications shown by the model BGRU+CNN+CRF are also evident in the model BLSTM+CNN+CRF. It has come to our attention that the model BLSTM+CNN+CRF has misclassified two additional tokens in sentences 4 and 5. In sentence 4, the word ঢাকা demonstrates contextual significance in its interpretation. Although it holds the potential to function as a location name, its usage in this particular sentence entails a distinct contextual meaning. The model BSTM+CNN+CRF incorrectly identifies it as a Location entity in this sentence. Moving on to sentence 5, the BLSTM+CNN+CRF model demonstrates an erroneous misclassification by incorrectly categorizing the idiomatic expression রাবণের চিতায় as a Location entity chunk. This misclassification highlights the model’s failure to accurately interpret the idiomatic nature of the phrase, consequently assigning it an incorrect semantic category.

## 7. Comparative analysis

The performance of NER systems vary with the amount of tokens used for training and testing, the tagging format, and the number of named entity classes. Although there has been some previous research on Bengali NER we were not able to fairly compare the performance of our proposed model with other existing models because our target named entity set, tagging scheme, and amount of training and testing tokens were different from all prior research. Moreover, we could not reproduce their models for our dataset because of the resources that other existing models rely on (i.e. word embedding, hand-crafted features, etc.) which are not available for public use. We can therefore only give a general overview of others’ work along with our work, see Tables 27 and 28.

**Table 27:** Results of other existing model along with our proposed model for Bengali NER.

Dataset	Models	Precision	Recall	F1
Bangla News Corpus	HMM [17]	79.48%	90.2%	84.5%
	SVM [21]	89.4%	94.3%	91.8%
	CRF [19]	87.8%	93.8%	90.7%
Anandabazar Patrika Corpus	NE Dictionary + Rule for NE + Left-Right Co-occurrence Statistics [24]	94.24%	85.50%	89.51%
IJCNLP-08 NERSSEAL Shared Task	Maximum Entropy (ME) [22]	82.63%	88.01%	85.22%
	Majority Voting Technique (ME, CRF, SVM) [25]	83.61%	87.11%	85.32%
	Weighted Voting Technique (ME, CRF, SVM-F, SVM-B) [23]	90.63%	93.98%	92.28%
	Margin Infused Relaxed Algorithm [26]	91.23%	87.29%	89.69%
Bangla Online Newspapers Dataset	GRU [27]	—	—	69.42%
Bangla Content Annotation Bank Named Entity Corpus	CRF [20]	Exact: 65%	Exact: 53%	Exact: 58%
		Partial: 78%	Partial: 67%	Partial: 72%
Bangla Newspaper Dataset	BGRU+CNN [28]	73.72%	72.27%	72.66%
Bangla Online News Sources and Banglapedia	DCN+BiLSTM [29]	68.95%	58.62%	63.37%
	BERT+BiLSTM+CRF+CW [30]	Macro: 65.60%	Macro: 66.78%	Macro: 65.96%
		Micro: 90.68%	Micro: 90.61%	Micro: 90.64%
Our Dataset	Our Proposed Model	Exact: 93.01%	Exact: 91.61%	Exact: 92.31%
		Partial: 88.85%	Partial: 86.19%	Partial: 87.50% Micro: 98.32%

**Table 28:** Dataset details of other existing models along with our proposed model for Bengali NER.

Entity	Study	Tag Scheme	Tag Set	Train Data	Test Data
Person, Location, Organization, Miscellaneous (Date, Time, Percentage, Monetary Expression)	[17] [21] [19]	BIE	17	150K (token)	10-fold cross validation
	[24]	BIE	17	70K (token)	12 test document [2592, 2938, 2477, 3816, 2944, 4843, 2899, 3420, 4428, 4228, 4528, 2991]
	[23]	BIE	17	150K (token)	30K (token)
	[22]	BIE	17	272K (token)	35K (token)
Person, Location, Organization, Miscellaneous	[25]	IJCNLP-08 NER shared task tag	12	1,22,467 (token)	10-fold cross validation
Person, Designation, Organization, Abbreviation, Brand, Title-Person, Title-Object, Location, Time, Number, Measure, Terms	[26]	IJCNLP-08 NER shared task tag	12	1,12,845 (token)	38,708 (token)
Person, Location, Organization, Day	[27]	IOB	9	—	—
Person, Location, Organization, Facility, Time, Units	[20]	IOB2	13	24,377 (token)	6,546 (token)
Person, Location, Organization, Time	[28]	IOB2	9	67,554 (token)	29,143 (token)
Person, Location, Organization, Object	[29]	IOB	9	—	—
	[30]	IOB	9	8,85,090 (token)	49,286 (token)
Person, Location, Organization, Quantity, Percentage, Currency	This Study	IOB2	13	8,04,637 (token)	2,01,154 (token)

## 8. Conclusions and future work

This research aims to improve Bengali NER while utilizing minimal resources by enhancing data quality. It has been proven that word embedding alone is insufficient for Named Entity Recognition in a morphologically enriched language like Bengali. Hence, we developed a robust system combining Bidirectional GRU, CNN and CRF that is capable of capturing morphologically complex Bengali words for NER tasks. Though the precision, recall and F1 scores of the models vary, word2vec (cbow) aided in obtaining optimal performance. Since our dataset is highly biased to the non-named entity class, we wrapped our models with focal loss and dice loss to address data imbalance and improve performance. Publicly available resources were used in the model development process so that other researchers can also utilize those resources with our best model for other sequence labelling tasks in Bengali and independently and advance Bengali Natural Language Processing research. Some of our goals for future work are as follows:

- In this research, the results for ‘B-ORG’ and idiomatic sentences were not satisfactory. Therefore, our dataset should be enriched with these kinds of samples and advanced techniques for accurately recognizing these entities will be explored.
- Our dataset will be enhanced with more data and data pre-processing in order to create a benchmark dataset for Bengali NER.
- Only non-contextualized word embeddings were used in this research. Experiments with contextualized word embeddings like BERT and its variants (DistilBERT, RoBERT) will be performed in the future.
- Data imbalances will be addressed with advanced techniques.
